# Enterohemorrhagic *E. coli* Requires N-WASP for Efficient Type III Translocation but Not for EspF_U_-Mediated Actin Pedestal Formation

**DOI:** 10.1371/journal.ppat.1001056

**Published:** 2010-08-19

**Authors:** Didier Vingadassalom, Kenneth G. Campellone, Michael J. Brady, Brian Skehan, Scott E. Battle, Douglas Robbins, Archana Kapoor, Gail Hecht, Scott B. Snapper, John M. Leong

**Affiliations:** 1 Department of Molecular Genetics and Microbiology, University of Massachusetts Medical School, Worcester, Massachusetts, United States of America; 2 Section of Digestive Diseases and Nutrition, University of Illinois at Chicago, Chicago, Illinois, United States of America; 3 Department of Medicine and Immunology, Massachusetts General Hospital, Boston, Massachusetts, United States of America; Institut Pasteur, France

## Abstract

Upon infection of mammalian cells, enterohemorrhagic *E. coli* (EHEC) O157:H7 utilizes a type III secretion system to translocate the effectors Tir and EspF_U_ (aka TccP) that trigger the formation of F-actin-rich ‘pedestals’ beneath bound bacteria. EspF_U_ is localized to the plasma membrane by Tir and binds the nucleation-promoting factor N-WASP, which in turn activates the Arp2/3 actin assembly complex. Although N-WASP has been shown to be required for EHEC pedestal formation, the precise steps in the process that it influences have not been determined. We found that N-WASP and actin assembly promote EHEC-mediated translocation of Tir and EspF_U_ into mammalian host cells. When we utilized the related pathogen enteropathogenic *E. coli* to enhance type III translocation of EHEC Tir and EspF_U_, we found surprisingly that actin pedestals were generated on N-WASP-deficient cells. Similar to pedestal formation on wild type cells, Tir and EspF_U_ were the only bacterial effectors required for pedestal formation, and the EspF_U_ sequences required to interact with N-WASP were found to also be essential to stimulate this alternate actin assembly pathway. In the absence of N-WASP, the Arp2/3 complex was both recruited to sites of bacterial attachment and required for actin assembly. Our results indicate that actin assembly facilitates type III translocation, and reveal that EspF_U_, presumably by recruiting an alternate host factor that can signal to the Arp2/3 complex, exhibits remarkable versatility in its strategies for stimulating actin polymerization.

## Author Summary

The food-borne pathogen enterohemorrhagic *E. coli* (EHEC) O157:H7 can cause severe diarrhoea and life-threatening systemic illnesses. During infection, EHEC attaches to cells lining the human intestine and injects Tir and EspF_U_, two bacterial molecules that alter the host cell actin cytoskeleton and stimulate the formation of “pedestals” just beneath bound bacteria. Pedestal formation promotes colonization during the later stages of infection. N-WASP, a host protein known to regulate actin assembly in mammalian cells, was previously shown to be manipulated by Tir and EspF_U_ to stimulate actin assembly, and to be required for EHEC to generate actin pedestals. Surprisingly, we show here that N-WASP promotes the efficient delivery of Tir and EspF_U_ into mammalian cells, and that when we utilized a related *E. coli* to enhance type III delivery of Tir and EspF_U_, actin pedestals assembled even in its absence. Thus, EHEC stimulates at least two pathways of actin assembly to generate pedestals, one mediated by N-WASP and one by an unidentified alternate factor. This flexibility likely reflects an important function of pedestal formation by EHEC, and study of the underlying mechanisms may provide new insights into the pathogenesis of infection as well as the regulation of the actin cytoskeleton of mammalian cells.

## Introduction

Enterohemorrhagic *Escherichia coli* (EHEC) are an important source of diarrheal illness worldwide and are the leading cause of pediatric renal failure in the United States. O157:H7 is the most common EHEC serotype associated with serious illness and includes many of the most virulent strains [1]. During colonization, EHEC induce striking morphological changes of the intestinal epithelium, resulting in the formation of attaching and effacing (AE) lesions. These structures are characterized by the effacement of microvilli and intimate attachment of EHEC to the epithelial cell surface. The adherent bacteria also reorganize the host cell cytoskeleton into filamentous (F-)actin pedestals. In addition to EHEC, several related pathogens, including enteropathogenic *E. coli* (EPEC), also generate AE lesions and actin pedestals on intestinal epithelial cells during the course of infection [1]. Importantly, mutations in any of these bacteria that abolish their ability to generate AE lesions prevent their colonization [2], [3], [4], [5]. Moreover, an EHEC mutant that is capable of intimate attachment but selectively defective for actin pedestal formation does not expand its initial infectious niche in experimentally-infected rabbits [6].

The capacity to generate actin pedestals depends on the translocation of bacterial effector proteins into mammalian host cells via a type III secretion system (T3SS) [7], [8]. This macromolecular structure spans the inner and outer bacterial membranes, extends from the bacterial surface, and includes a long filamentous appendage that contacts the mammalian cell surface and functions as a conduit for effector secretion. The tip of this filament includes translocator proteins that form pores in target cell membranes and promote the entry of effectors into the mammalian cell.

The EHEC- and EPEC-encoded type III secretion apparatuses are homologous to the T3SSs found in a wide range of pathogens, many of which also trigger actin rearrangements in the host cell. For example, type III translocated effectors of *Shigella*, *Salmonella*, and *Yersinia* induce cytoskeletal changes that can promote bacterial entry into the host cell. Actin assembly may also affect type III translocation, because several effectors that misregulate signaling pathways that control the actin cytoskeleton have a significant influence on the efficiency of translocation by *Shigella* and *Yersinia* [9], [10].

For AE pathogens, the T3SS delivers effectors that activate the WASP and N-WASP actin nucleation-promoting factors to promote pedestal formation [11], [12], [13]. WASP, which is expressed in hematopoietic cells, and its homolog N-WASP, which is ubiquitously expressed, stimulate the Arp2/3 complex, a group of seven proteins that collectively nucleate actin into filaments [14], [15]. The C-terminal WCA (WH2-connector-acidic) domain of N-WASP directly binds and activates the Arp2/3 complex, but this domain is normally sequestered by its intramolecular interaction with an internal regulatory element, the GBD (GTPase-binding l;domain). Binding of the GTPase Cdc42 to the GBD disrupts these autoinhibitory GBD-WCA interactions, and frees the WCA domain to activate Arp2/3-mediated actin assembly. Other factors, including the SH2/SH3 domain-containing adaptor proteins Nck1-2, also activate N-WASP, but bind to a proline-rich domain (PRD) that lies between the GBD and WCA regions [16], [17].

One effector essential for intimate attachment and actin pedestal formation by AE pathogens is the Tir (translocated intimin receptor) protein [18], [19]. Upon type III translocation into the mammalian cell, Tir becomes localized in the plasma membrane with a central extracellular domain that binds the bacterial outer membrane adhesin intimin [20]. N- and C-terminal to the intimin-binding domain are two transmembrane segments and the intracellular domains of Tir. For canonical EPEC strains of serotype O127:H6, Tir is the only effector required for pedestal formation, as simply clustering Tir in the plasma membrane is sufficient to recruit the Nck adaptor proteins and trigger F-actin assembly [21].

In contrast to canonical EPEC strains, EHEC strains of serotype O157:H7 require a second translocated effector, in addition to Tir, to trigger pedestal formation. EHEC Tir recruits this effector, named EspF_U_ (also known as TccP) [22], [23], indirectly, as the host protein intermediates IRTKS and IRSp53 are responsible for linking EspF_U_ to Tir during actin pedestal assembly [24], [25]. EspF_U_ contains a C-terminal region with multiple 47-residue proline-rich repeats that each bind to the GBD of N-WASP and directly displace the WCA domain to allow it to activate the Arp2/3 complex [26], [27]. Whereas a single EspF_U_ repeat is capable of activating N-WASP, tandem repeats synergize during actin polymerization by promoting N-WASP dimerization, which allows it to bind Arp2/3 with much higher affinity than monomeric N-WASP [27], [28], [29].

EHEC is unable to generate pedestals on N-WASP-deficient cells [12], and the fact that EspF_U_ targets N-WASP to promote actin assembly provides a highly plausible explanation for this finding. Nevertheless, the observations that actin assembly influences type III translocation by other pathogens raised the possibility that N-WASP may also contribute to an earlier step in the process of pedestal formation. In fact, we show here that N-WASP and actin assembly are important for the translocation of Tir and EspF_U_ into mammalian cells by EHEC O157:H7. Intriguingly, when delivered into cells by EHEC-independent means, Tir and EspF_U_ are fully capable of stimulating actin pedestal formation in the absence of N-WASP. These results add an additional layer of complexity to our understanding of the interactions between EHEC and its host cells, and highlight the functional versatility of EspF_U_.

## Results

### N-WASP-mediated actin assembly facilitates type III translocation of Tir and EspF_U_ from EHEC

**Figure 1:**
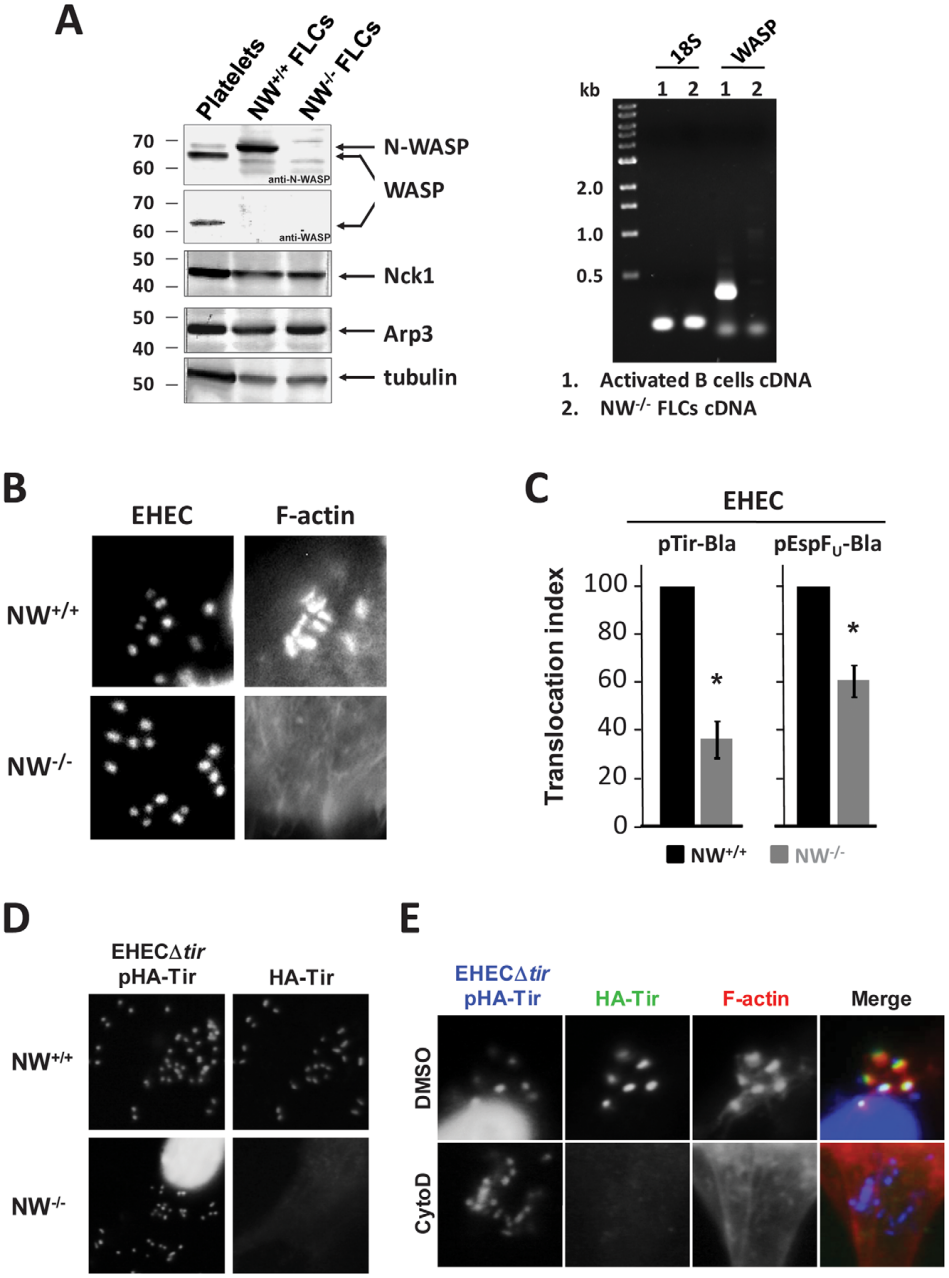
N-WASP-mediated actin assembly facilitates type III translocation of Tir and EspF_U_ from EHEC. (**A**) Extracts from platelets, N-WASP positive fibroblast-like cells (“NW^+/+^ FLCs”) or N-WASP knockout (NW^−/−^) FLCs were resolved by SDS-PAGE and immunoblotted for N-WASP, WASP, Nck1, Arp3 or tubulin (Left). RT-PCR analysis of WASP mRNA was performed for RNA extracted from NW^−/−^ cells or activated B cells (Right). (**B**) NW^+/+^ or NW^−/−^ cells were infected with EHEC and examined after staining with DAPI to localize bacteria and Alexa568-phalloidin to detect F-actin. (**C**) Translocation of a Tir-Bla or EspF_U_-Bla fusion by EHEC in NW^+/+^ and NW^−/−^ cells was measured by detecting cleavage of the β-lactamase FRET reporter CCF2-AM, which results in a change in fluorescent emission of cells from green (absence of detectable Tir-Bla) to blue (presence of Tir-Bla) [31]. Monolayers were infected for 6 hours, incubated with CCF2-AM, and fixed. The percentage of blue cells was scored visually by fluorescent microscopy to determine the translocation index. Shown is the mean ± SD of three experiments; *P<0.05. (**D**) NW^+/+^ and NW^−/−^ FLCs were infected with EHECΔ*tir* harboring plasmid pHA-Tir for 5 hours and examined after staining with DAPI to detect bacteria, anti-HA antibody to detect Tir foci and Alexa568-phalloidin for detection of F-actin. (**E**) DMSO- or cytochalasin D-treated HeLa cells were infected with EHECΔ*tir* harboring plasmid pHA-Tir, fixed, and stained with DAPI to detect bacteria, anti-HA antibody to visualize Tir foci and Alexa568-phalloidin to detect F-actin.

N-WASP deficiency in cultured mammalian cells is known to block actin pedestal formation by EHEC [12]. An obvious rationale for this requirement is that N-WASP promotes actin polymerization in the pedestal, as suggested by the observation that EspF_U_ recruits, binds and activates N-WASP [22], [23]. However, given the evidence that actin polymerization might also facilitate the delivery of effectors into the host cell [9], [10], we examined a role for N-WASP during type III effector translocation by EHEC using genetically modified murine fibroblast-like cells (FLCs) [30]. Consistent with the previous characterization of wild type (NW^+/+^) and N-WASP knockout (NW^−/−^) cell lines, immunoblotting demonstrated that N-WASP was expressed only in the wild type cells (Figure 1A, left). We also investigated the expression of the N-WASP homolog WASP, which is also a target of EspF_U_ [26], and found that neither WASP mRNA or protein was detected in NW^−/−^cells (Figure 1A, right). As reported using an independently derived N-WASP-deficient cell line [12], EHEC generated actin pedestals on wild type, but not knockout cells (Figure 1B).

To assess Tir translocation, we fused the C-terminus of the EHEC Tir molecule to the TEM-1 β-lactamase (Bla). The translocation of this fusion protein into host cells can be detected by β-lactamase-mediated cleavage of a FRET reporter, resulting in a change in fluorescent wavelength emission from green (520 nm) to blue (460 nm), as previously described [31]. Such fusions have been used extensively for assessing Tir translocation [32], and maintain Tir function, as our Tir-Bla fusion complemented a bacterial Tir deletion for pedestal-forming function on NW^+/+^ cells (Figure S1A). After infection of wild type or N-WASP-deficient FLCs with EHEC expressing the Tir-Bla fusion, the percentage of blue cells was scored visually by fluorescent microscopy (Figure S1B) and expressed as a translocation index. By this measure, the translocation of Tir by EHEC into N-WASP-knockout cells occurred ∼3-fold less efficiently than into wild type cells after a 6h infection (Figure 1C). The requirement for N-WASP for efficient translocation was not restricted to Tir, because the level of translocation of an EspF_U_-Bla fusion into N-WASP-knockout cells was also diminished relative to wild type cells, albeit not quite as low as translocation of Tir-Bla (Figure 1C). In accordance with these results, we found that treatment of HeLa cells with wiskostatin, an inhibitor of N-WASP, significantly impaired translocation of the EspF_U_-Bla fusion into host cells (Figure S1C).

Given that the Tir-Bla translocation index relies on binary scoring of (green vs. blue) cells by visual inspection, it may not reflect the true severity of the defect in Tir translocation into NW^−/−^ cells. The deficiency in the translocation of Tir into N-WASP-deficient cells by EHEC is predicted to result in a decrease in the amount of Tir clustered beneath bound bacteria. Therefore, to examine Tir localization, we infected wild type or knockout cells with EHECΔ*tir* harboring pHA-Tir_EHEC_, which encodes an N-terminally HA-tagged Tir that can be detected with an anti-HA antibody and visualized microscopically. Whereas Tir foci were readily observed beneath EHEC bound to wild type cells, they were not detected beneath EHEC on N-WASP-knockout cells (Figure 1D), consistent with a significant defect in Tir translocation.

To test whether the requirement for N-WASP for efficient translocation reflects a role for F-actin assembly in promoting translocation, we examined Tir localization beneath bound bacteria after treatment with cytochalasin D, which binds actin filament ends and prevents polymerization. Microscopic visualization revealed that cytochalasin D treatment resulted in a loss of foci of HA-tagged Tir beneath bacteria bound to HeLa cells (Figure 1E). In addition, cytochalasin D and latrunculin A, which binds actin monomers and triggers depolymerization, each partially inhibited of translocation of Tir-Bla fusion protein into HeLa cells (data not shown). Collectively, these data suggest that N-WASP-mediated actin polymerization facilitates EHEC-mediated effector translocation.

### N-WASP is required for efficient intimin-mediated bacterial attachment to the cell surface

**Figure 2:**
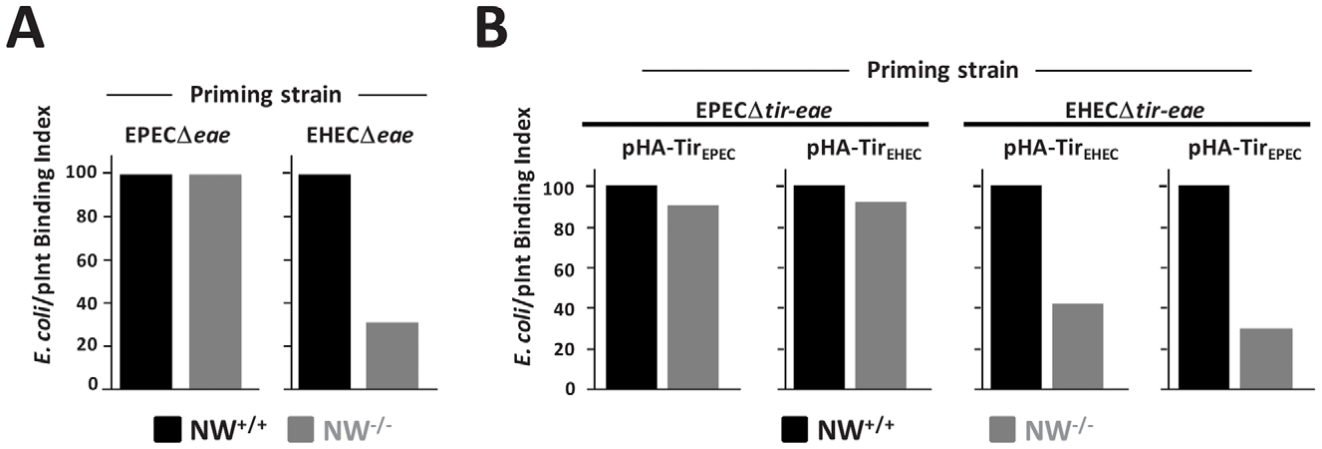
N-WASP is required for efficient intimin-mediated bacterial attachment to the cell surface. (**A**) A so-called “prime and challenge” assay was utilized to evaluate Tir/intimin-mediated binding of intimin-expressing *E. coli* to NW^+/+^ or NW^−/−^ FLCs. Cells were infected with EPECΔ*eae* or EHECΔ*eae* strains to allow for translocation Tir. After gentamicin treatment to kill EPEC and EHEC, Tir_EPEC_- and Tir_EHEC_-“primed” cells were “challenged” (i.e. infected) for 1 hour, respectively with *E. coli* K12/pInt_EPEC_ or K12/pInt_EHEC_ that also harbor a plasmid encoding GFP and examined microscopically to detect bound bacteria. The bacterial binding index was defined as the percentage of cells with at least five bound bacteria. (**B**) NW^+/+^ or NW^−/−^ cells were infected with EPECΔ*tir-eae* or EHECΔ*tir-eae* harboring either pHA-Tir_EPEC_ or pHA-Tir_EHEC_ and the bacterial binding index determined as described as above. The experiments described in **A** and **B** were performed two times; data from one representative experiment are presented.

We next tested whether impaired Tir translocation into N-WASP-knockout FLCs results in a measurable effect on the ability of Tir to promote bacterial attachment. We infected wild type or N-WASP-knockout cells with the intimin-deficient EPECΔ*eae* or EHECΔ*eae* mutants to allow for translocation of Tir, and then, after killing these bacteria with gentamicin and removing them by washing, challenged these cells with non-pathogenic GFP-expressing *E. coli* strains that harbor pInt_EPEC_ or pInt_EHEC_ plasmids to express intimin. Previous studies have shown that *E. coli*/pInt, but not *E. coli*/vector, attach to monolayers primed with EPEC or EHEC strains that translocate Tir, but not to unprimed monolayers [33], [34], thus allowing a specific measure of native intimin binding to translocated Tir. A bacterial binding index, defined as the percentage of cells with at least five adherent GFP- and intimin-expressing bacteria, was determined microscopically. Bacterial binding to N-WASP-knockout cells primed with EHECΔ*eae* was approximately 3-fold lower than to primed wild type cells (Figure 2A).

EPEC generates pedestals on cultured cells more efficiently than EHEC [35], so we tested whether EPEC might correspondingly translocate Tir into N-WASP-knockout cells more efficiently. In fact, bacterial binding to N-WASP-deficient cells primed with EPECΔ*eae* was indistinguishable from binding to EPECΔ*eae*-primed wild type cells (Figure 2A). To test whether the difference between EHEC and EPEC in functional Tir translocation was due to allelic differences in their respective Tir proteins, we primed wild type or N-WASP-knockout FLCs with EPECΔ*tir-eae* expressing either HA-Tir_EPEC_ or HA-Tir_EHEC_, and then challenged cells with *E. coli* expressing the corresponding intimin ligand. Alternatively, we primed cells with EHECΔ*tir-eae* expressing either HA-Tir_EHEC_ or HA-Tir_EPEC_ prior to challenge. We found that EPEC was capable of translocating either Tir variant into N-WASP-knockout cells to promote intimin-mediated attachment at nearly wild type levels. In contrast, priming with EHEC expressing either HA-Tir_EHEC_ or HA-Tir_EPEC_ gave binding values two- to three-fold lower than wild type (Figure 2B). These observations indicate that Tir translocation by EHEC is more dependent on N-WASP than Tir translocation by EPEC, irrespective of the genetic origin of the Tir molecule.

### Tir_EHEC_ and EspF_U_ promote actin pedestal formation in the absence of N-WASP

**Figure 3:**
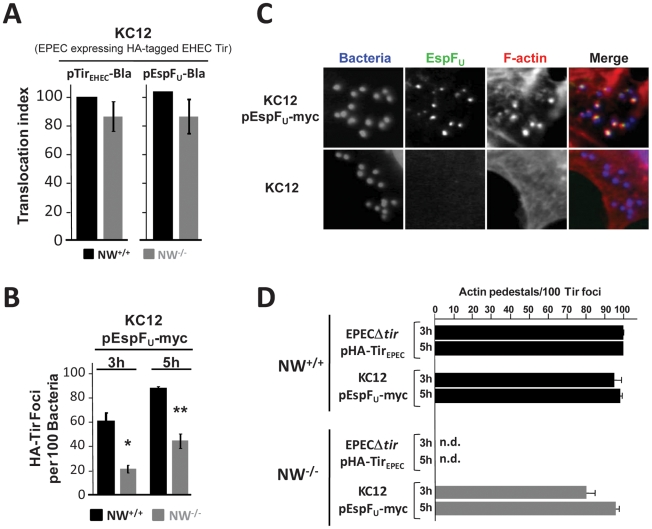
Tir_EHEC_ and EspF_U_ promote pedestal formation in the absence of N-WASP. (**A**) Translocation of the Tir_EHEC_-Bla and EspF_U_-Bla fusions by KC12 in NW^+/+^ and NW^−/−^ FLCs was measured 6 hours postinfection in the TEM-1 β-lactamase translocation assay as described in Figure 1. Shown is the mean ± SD of three experiments. (**B**) NW^+/+^ and NW^−/−^ cells were infected with KC12/pEspF_U_-myc, and the percentage of HA-Tir foci associated with bound bacteria was determined at 3 and 5 hours postinfection. Values represent the mean ± SD of three experiments; *P<0.05; **P<0.01. (**C**) NW^+/+^ and NW^−/−^ cells were infected with KC12 or KC12/pEspF_U_-myc and examined after staining with DAPI, anti-myc antibody and Alexa568-phalloidin. (**D**) NW^+/+^ or NW^−/−^ cells were infected with EPECΔ*tir*/pHA-Tir_EPEC_ or KC12/pEspF_U_-myc, and then fixed, and stained with DAPI, anti-HA antibody to visualize Tir foci and Alexa568-phalloidin. To quantitate pedestal formation, the percentage of actin pedestals that colocalized with HA-Tir foci-associated bacteria at 3 and 5 hours postinfection was determined visually by fluorescent microscopy (right panel). Data represent the mean ± SD from three experiments. “n.d.”; not detected.

The observations that EHEC does not efficiently translocate Tir or EspF_U_ into N-WASP-deficient cells, raised the intriguing possibility that the defect in EHEC pedestal formation on these cells was due to inefficient effector translocation into cells rather than a lack of Tir-EspF_U_ signaling within the cell. Since, in the functional assay described above, EPEC translocated Tir into N-WASP knockout cells better than EHEC, we adopted a heterologous expression system using KC12, an EPEC derivative that has been chromosomally engineered to express HA-tagged EHEC Tir [22], [36], for achieving delivery of EHEC Tir and EspF_U_ into N-WASP-knockout cells. Importantly, although translocation of Tir_EHEC_-Bla and EspF_U_-Bla into N-WASP-deficient cells by KC12 occurred with somewhat delayed kinetics compared to wild type cells (Figure S2), the defect in translocation was mild at 6 h postinfection (Figure 3A). To determine if type III translocation by KC12 was reflected in the localization of Tir beneath bound bacteria, we infected N-WASP-knockout cells with KC12/pEspF_U_, a strain that expresses a myc-tagged EspF_U_ harboring six C-terminal repeats and generates actin pedestals in manner that is mechanistically indistinguishable from canonical EHEC strains [22]. HA-Tir foci were observed with somewhat delayed kinetics and lower frequency in NW^−/−^ than NW^+/+^ FLCs, but nearly 50% of KC12/pEspF_U_ bound to N-WASP-knockout cells generated Tir foci by 5 h postinfection (Figure 3B).

Given that KC12/pEspF_U_ was only partially diminished for Tir and EspF_U_ translocation, we sought to determine whether this strain could generate actin pedestals on N-WASP knockout cells. Remarkably, upon infection of NW^−/−^ FLCs, numerous actin pedestals were formed by KC12/pEspF_U_ (Figure 3C, top row), indicating that EHEC Tir and EspF_U_ are capable of signaling to the actin cytoskeleton in the absence of N-WASP. Pedestal formation required EspF_U_, because KC12 lacking pEspF_U_ failed to generate pedestals in these cells (Figure 3C, bottom row). To quantify the efficiency of actin pedestal formation, we infected wild type and N-WASP-knockout cells with KC12/pEspF_U_, visually identified sites of HA-Tir localization beneath bound bacteria, and then calculated the percentage of those Tir foci that were associated with actin pedestals. This specific scoring method circumvented the inhibitory effects of N-WASP deficiency on effector entry (Figure 1C; Figure 2) and HA-Tir localization in cells (Figure 3B), and specifically measured intracellular signaling after Tir translocation. KC12/pEspF_U_ and the control strain EPECΔ*tir*/pHA-Tir_EPEC_, which generates pedestals using the Nck-N-WASP-dependent pathway [13], [36], [37], both formed pedestals efficiently on wild type cells: after infection for 3h, >95% of Tir foci were associated with pedestals, while at 5h this level reached >98% (Figure 3D). In NW^−/−^ FLCs, EPECΔ*tir*/pHA-Tir_EPEC_, which utilizes Nck adaptor proteins to activate N-WASP, was totally incapable of generating pedestals (Figure 3D), consistent with results utilizing an independently generated N-WASP knockout cell line [13]. In contrast, 80% of KC12/pEspF_U_-associated Tir foci triggered actin pedestals at 3h, and this level rose to 95% at 5h postinfection. Thus, the more efficient delivery of EHEC Tir and EspF_U_ by the EPEC-derived strain KC12 results in a surprisingly effective ability to induce pedestal formation in the absence of N-WASP.

### The Tir-EspF_U_ linker protein IRTKS localizes to bacteria in the absence of N-WASP

**Figure 4:**
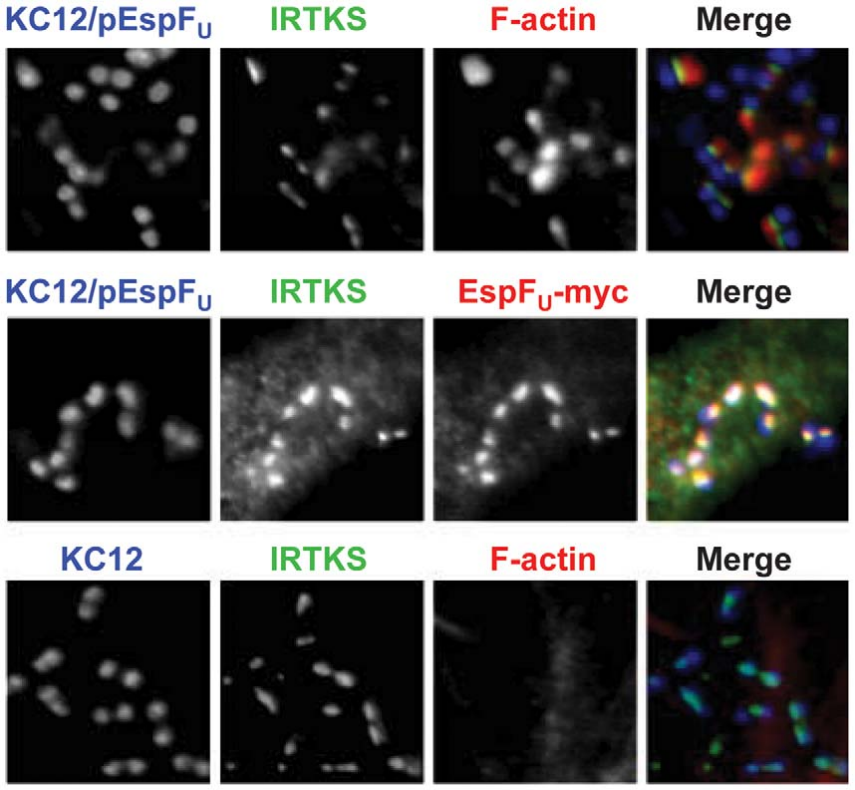
The Tir-EspF_U_ linker protein IRTKS localizes to bacteria even in the absence of N-WASP. NW^−/−^ FLCs were infected with KC12/pEspF_U_ or KC12, and examined after staining with DAPI to detect bacteria, anti-IRTKS antibody (green) and Alexa568-phalloidin to detect F-actin (red). To detect EspF_U_-myc, monolayers were treated with rabbit anti-myc antibody and Alexa568-conjugated anti-rabbit antibody (red, middle row).

IRTKS, along with the closely related protein IRSp53, regulates actin dynamics at the plasma membrane [38], and functions as a linker between EHEC Tir and EspF_U_ during N-WASP-promoted pedestal formation [24], [25]. Given that EspF_U_ localized to sites of bacterial attachment in N-WASP-knockout cells (Figure 3C), we assessed whether IRTKS plays a role in EspF_U_ recruitment in the absence of N-WASP by examining the distribution of IRTKS in N-WASP-knockout cells infected with KC12/pEspF_U_. Immunofluorescence microcopy indicated that IRTKS localized near the tips of pedestals (Figure 4, top row), where it colocalized with EspF_U_ (middle row), consistent with a role in linking Tir and EspF_U_ during N-WASP-independent signaling. Moreover, when these cells were infected with KC12 lacking EspF_U_, IRTKS still localized to sites of bacterial attachment, suggesting that even in the absence of EspF_U_, N-WASP, and actin pedestals, the Tir-binding activity of IRTKS is sufficient to promote IRTKS recruitment (Figure 4, bottom row). Thus, N-WASP does not have any apparent effects on the signaling events that occur between type III effector translocation and EspF_U_ recruitment to Tir.

### Clustering EspF_U_ at the plasma membrane is sufficient to drive N-WASP independent pedestal formation

**Figure 5:**
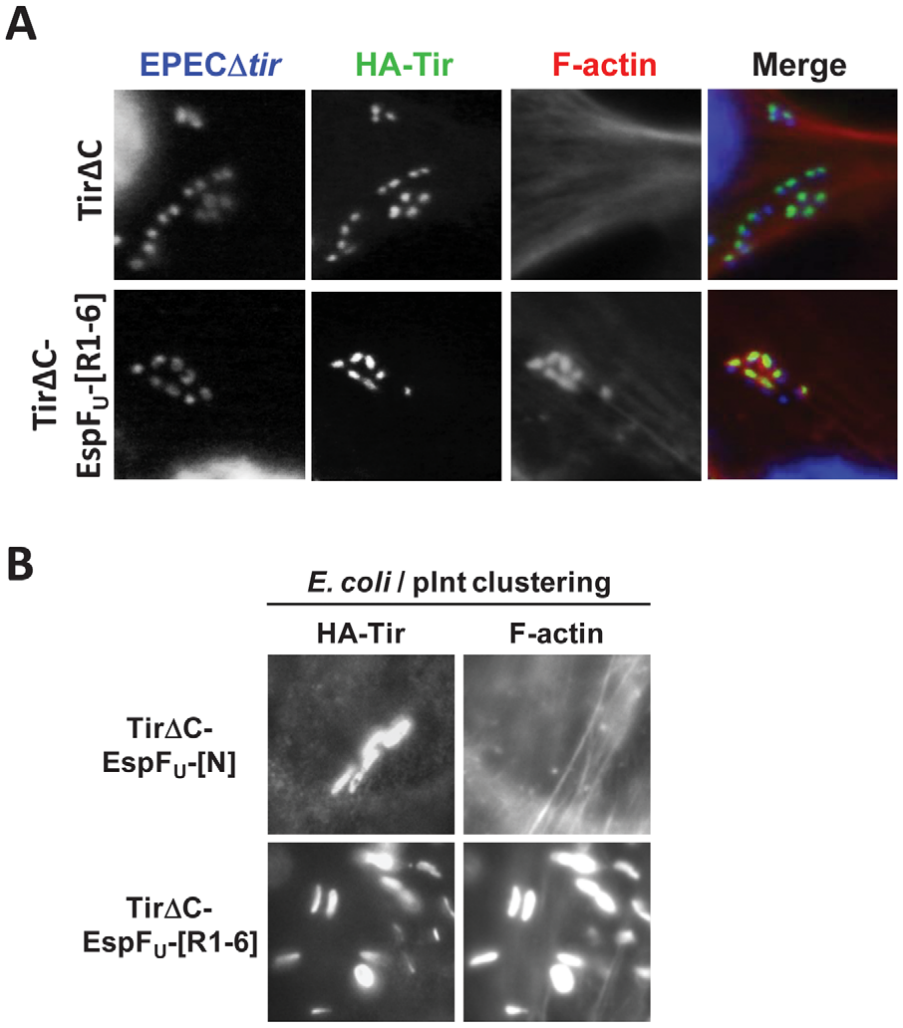
EspF_U_ is necessary and sufficient to drive N-WASP independent pedestal formation. (**A**) NW^−/−^ FLCs ectopically expressing HA-Tir derivatives in which the C-terminal cytoplasmic domain was deleted (TirΔC) or replaced with the C-terminal repeats of EspF_U_ (TirΔC-EspF_U_-[R1-6]) were challenged with an EPECΔ*tir* strain (which expresses intimin), and stained with DAPI (blue), anti-HA antibody (green) and Alexa568-phalloidin (red). (**B**) NW^−/−^ FLCs ectopically expressing HA-Tir derivatives in which the C-terminal cytoplasmic domain was replaced with the N-terminal translocation sequence (TirΔC-EspF_U_-[N]) as a negative control or the C-terminal repeats of EspF_U_ (TirΔC-EspF_U_-[R1-6]) were challenged with *E. coli*/pInt to cluster the fusion protein, and treated as in **A**.

For pedestal formation by EHEC on wild type cells, recruitment and membrane clustering of a complex of Tir, IRTKS and EspF_U_ is sufficient to trigger pedestal formation [24]. However, we have also shown that membrane clustering of HN-Tir-EspF_U_-[R1-6], a fusion in which the C-terminal cytoplasmic domain of Tir is replaced by six C-terminal repeats of EspF_U_, is fully functional for pedestal formation [26], [28], indicating that clustering of EspF_U_ alone is sufficient to stimulate this signaling pathway. To similarly determine the potential requirements of EspF_U_, Tir and IRTKS during N-WASP-independent actin pedestal formation, we tested whether HN-Tir-EspF_U_-[R1-6] could trigger actin assembly in N-WASP-knockout cells. After transfection with a plasmid encoding HN-Tir-EspF_U_-[R1-6], we infected NW^−/−^ FLCs with EPECΔ*tir* and treated cells with an anti-HA antibody to visualize the fusion protein and with phalloidin to stain F-actin. These bacteria readily generated actin pedestals on cells expressing HN-Tir-EspF_U_-[R1-6], but not cells expressing HN-TirΔC, which lacks a C-terminal signaling domain (Figure 5A), indicating that the EspF_U_ repeats are essential for actin pedestal formation in these cells.

To test whether pedestal formation on N-WASP-knockout cells requires any proteins other than the Tir-EspF_U_ fusion, we next treated HN-Tir-EspF_U_-[R1-6]-expressing cells with the non-pathogenic *E. coli* strain that expresses intimin. These bacteria, which are incapable of type III secretion and serve to simply cluster the HN-Tir-EspF_U_-[R1-6] fusion protein, generated actin pedestals on N-WASP-knockout cells in a manner indistinguishable from those formed on wild type cells (Figure 5B and [26], [28]). In contrast, a control HN-Tir fusion protein lacking the C-terminal repeats of EspF_U_ was unable to elicit pedestals. Thus, we conclude that, as is the case for pedestal formation in N-WASP-proficient cells, the central role of Tir and IRTKS in N-WASP-knockout cells is to promote the clustering of the EspF_U_ repeats beneath the plasma membrane. Moreover, in the absence of N-WASP, EspF_U_ remains the most essential component of the signaling pathway that leads to actin pedestal assembly.

### The Arp2/3 complex is critical for actin pedestal formation on N-WASP-deficient cells

**Figure 6:**
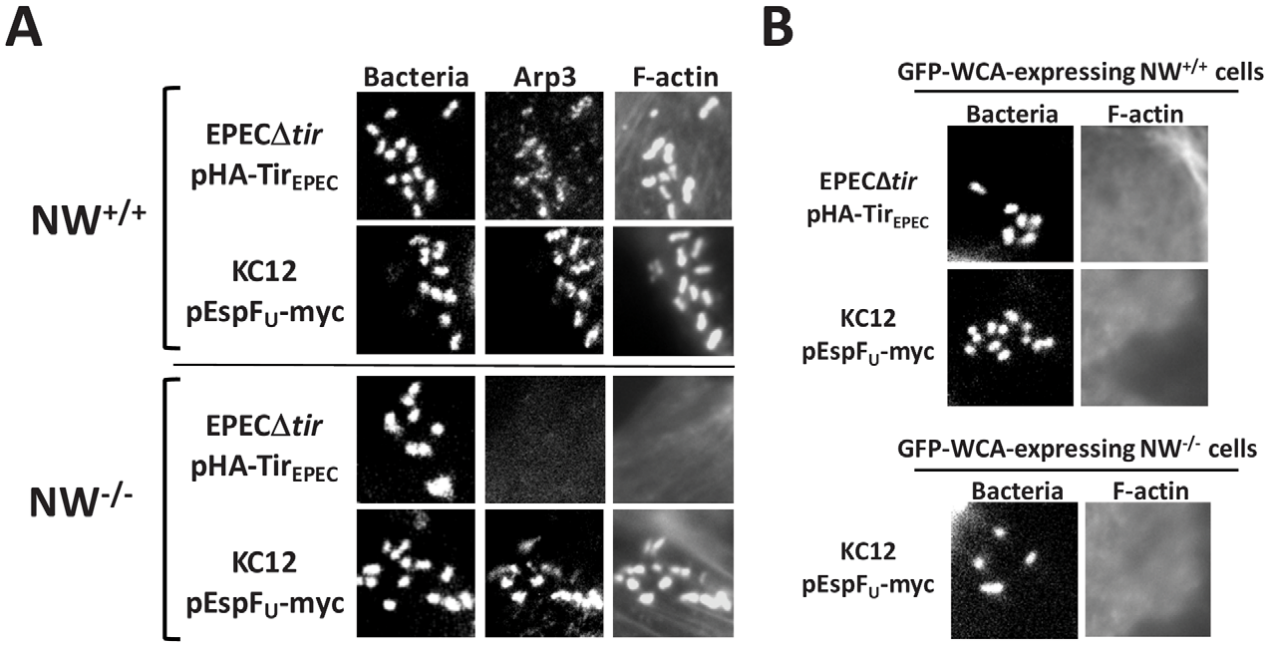
The Arp2/3 complex is critical for actin pedestal formation on N-WASP-deficient cells. (**A**) NW^+/+^ or NW^−/−^ FLCs were infected with KC12/pEspF_U_ or EPECΔ*tir*/pHA-Tir_EPEC_, fixed, and stained with DAPI to detect bacteria, anti-Arp3 antibody to visualize the Arp2/3 complex and Alexa568-phalloidin to detect F-actin. (**B**) Transfected NW^+/+^ or NW^−/−^ cells expressing GFP fused to the WCA domain of N-WASP (GFP-WCA) were infected with KC12/pEspF_U_ or EPECΔ*tir*/pHA-Tir_EPEC_. Monolayers were stained with DAPI and Alexa568-phalloidin, and transfected cells were identified by GFP fluorescence. In two independent experiments, expression of the GFP-WCA fusion protein strongly inhibited pedestal formation.

The interaction of EspF_U_ with N-WASP or WASP results in the activation of the Arp2/3 complex and actin nucleation *in vitro* [22], [26], [27], [39]. To examine the potential role of Arp2/3 in pedestals generated in the absence of N-WASP, we first assessed whether this complex is recruited to sites of pedestal formation in N-WASP-knockout cells. Immunofluorescence microscopy using anti-Arp3 antibodies revealed recruitment of the Arp2/3 complex in pedestals formed by KC12/pEspF_U_ in N-WASP knockout cells as well as wild type cells (Figure 6A), suggesting that Arp2/3 is likely involved in actin pedestal formation under both circumstances.

To test for a functional role of the Arp2/3 complex in pedestal formation, we took advantage of the fact that overexpression of the N-WASP WCA domain results in sequestration and/or ectopic activation of the Arp2/3 complex [14], [15], [40]. Whereas 95% of cells expressing a GFP control protein contained pedestals upon infection with EPECΔ*tir*/pHA-Tir_EPEC_ or KC12/pEspF_U_, <5% of wild type FLCs expressing GFP-WCA harbored pedestals (Figure 6B and data not shown), confirming the importance of proper Arp2/3 activity in actin pedestal assembly. Moreover, this dominant negative GFP-WCA construct also blocked actin pedestal formation by KC12/pEspF_U_ in N-WASP-knockout FLCs (Figure 6B). Finally, genetic depletion of the Arp2/3 subunits Arp3 and ARPC4 abolished pedestal formation on wild type HeLa cells, which are predicted to support both N-WASP-dependent and N-WASP-independent pedestal formation (Figure S3). Consistent with previous reports, we found that EspF_U_ derivatives were unable to directly activate the Arp2/3 complex to promote actin polymerization *in vitro* (Figure S4; [23], [27]). Collectively, these data suggest that in generating pedestals in N-WASP-deficient cells, EspF_U_ recruits an alternate host factor (or factors) that triggers Arp2/3-mediated actin assembly.

### EspF_U_ does not recruit other previously characterized members of the WAVE/WASP family

**Figure 7:**
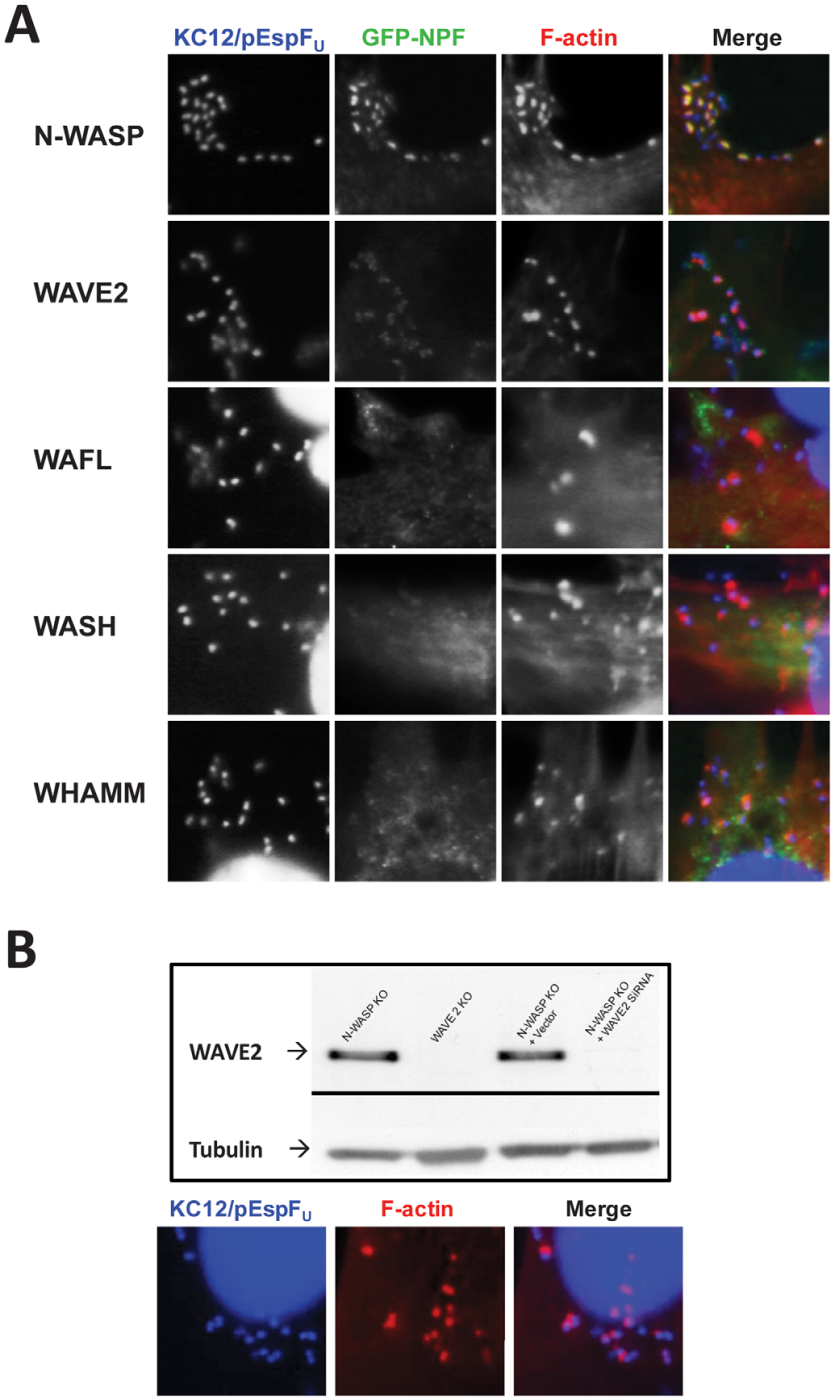
WASP/WAVE family members that are not involved in pedestal formation. (**A**) NW^−/−^ fibroblast-like cells ectopically expressing GFP fusions to WASP/WAVE family members N-WASP, WAVE2, WASH, or WHAMM, or to WAFL, were infected with EPEC KC12/pEspF_U_, and examined after staining with DAPI to detect bacteria (blue) and Alexa568-phalloidin to visualize F-actin (red). (**B**) Genetic depletion of WAVE2 in N-WASP knockout cells does not affect pedestal formation. Extracts from NW^−/−^ cells, WAVE2^−/−^ cells, or NW^−/−^ cells harboring control vector or one expressing a WAVE2 siRNA, were resolved by SDS-PAGE and immunoblotted for WAVE2 or tubulin, as a loading control (Top panel). NW^−/−^/WAVE2 knockdown cells were infected with KC12/pEspF_U_, and examined after staining with DAPI to detect bacteria and Alexa568-phalloidin to visualize F-actin (Bottom panel).

WASP and N-WASP are members of a family of nucleation promoting factors (NPFs) that activate Arp2/3, a family that includes WAVE proteins, WASH, and WHAMM [41]. IRSp53, which has been shown to link Tir and EspF_U_ in some cells [25] can bind and activate WAVE2 [38]. In addition, WAFL is a protein with a predicted Arp2/3-binding acidic peptide that associates with actin filaments and has been implicated in endosomal trafficking [42]. To investigate whether these factors could be involved in N-WASP-independent actin pedestal formation, we determined whether they localized to actin pedestals generated in an N-WASP-independent manner. NW^−/−^ FLCs ectopically expressing GFP fusions to WAVE2, WASH, WAFL, WHAMM, or (as a control) N-WASP were infected with KC12/pEspF_U_ and phalliodin-stained to visualize actin pedestals. Pedestals were efficiently formed in the presence of all NPFs, and as expected, GFP-N-WASP distinctly localized to pedestals (and often to their tips) beneath bound bacteria (Figure 7A, top row, and data not shown). None of the other NPFs localized in a similar fashion (Figure 7A). GFP-WAVE2 faintly and diffusely localized to sites of bacterial attachment (Figure 7A, second row), but this localization was also observed around bacteria that were not associated with actin pedestals (data not shown). Furthermore, WAVE2 was not required for N-WASP-independent pedestal formation, because KC12/pEspF_U_ generated pedestals normally on NW^−/−^ FLCs in which WAVE2 expression was stably knocked down by more than 95% (Figure 7B). Together with the observation that EspF_U_ does not directly activate Arp2/3, these data are consistent with the model that EspF_U_ is capable of utilizing an alternate NPF to activate Arp2/3 in NW^−/−^ FLCs.

### N-WASP-independent actin pedestal formation requires multiple EspF_U_ repeats

**Figure 8:**
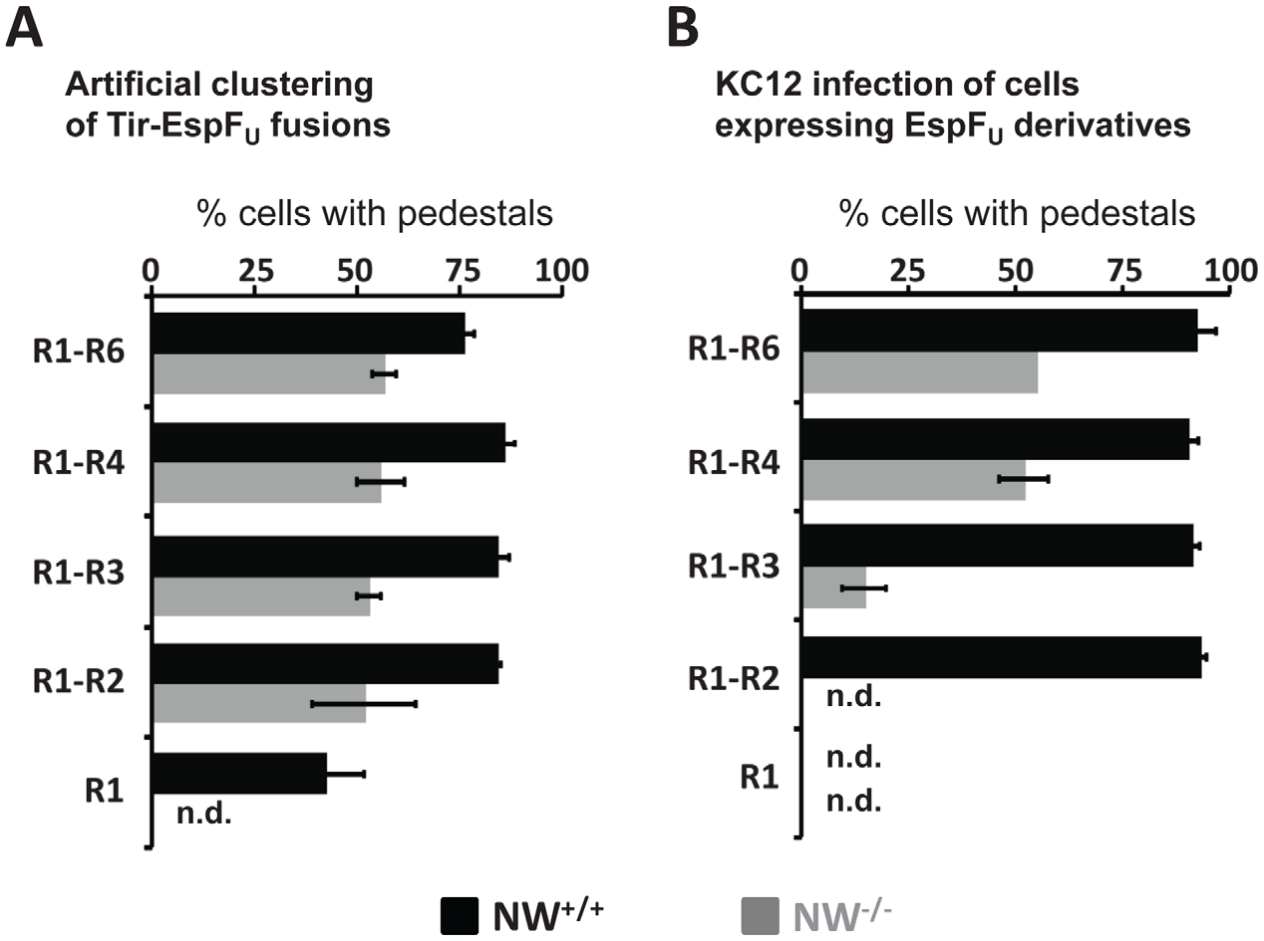
Pedestal formation on N-WASP-deficient cells requires multiple EspF_U_ repeats. (**A**) NW^−/−^ cells (shaded bars) ectopically expressing Tir-EspF_U_ fusion derivatives were treated with anti-Tir antibodies and *S. aureus* particles to promote membrane clustering. Monolayers were stained with anti-HA antibody to identify both transfected cells and *S. aureus* (which binds the fluorescent antibodies) and with Alexa568-phalloidin to detect F-actin. The pedestal formation indices were determined by calculating the percentage of transfected cells harboring five or more *S. aureus* particles associated with actin pedestals. These experiments were performed in parallel with NW^+/+^ cells (solid bars), the results of which were previously published [28] and are shown here for comparison. Data represent the mean ± SD from three experiments. (**B**) NW^−/−^ (shaded bars) ectopically expressing GFP-EspF_U_ fusion derivatives were infected with EPEC KC12 and monolayers stained with DAPI to identify attached bacteria, anti-myc antibody to enhance detection of GFP-EspF_U_ fusion and Alexa568-phalloidin to detect F-actin. The pedestal formation indices were determined by calculating the percentage of transfected cells harboring five or more actin pedestals. These experiments were performed in parallel with NW^+/+^ (solid bars) cells, the results of which were previously published [28] and are shown here for comparison. Data represent the mean ± SD from three experiments. “n.d.”; not detected.

Allosterically activated N-WASP is associated with more potent actin assembly when multimerized [29], [43], [44], [45], an observation explained by the ability of dimeric N-WASP to bind the Arp2/3 complex with much higher affinity than monomeric N-WASP [29]. Nevertheless, when Tir-EspF_U_ fusions harboring different numbers of repeats are clustered beneath the plasma membrane using anti-Tir antibody-coated particles, a single EspF_U_ repeat is capable of triggering actin pedestal formation in N-WASP-proficient cells [26], [28]. This prompted us to examine the role of the repeat quantity in N-WASP-independent actin assembly. To directly compare a requirement for different numbers of repeats during EspF_U_-mediated assembly in the presence or absence of N-WASP, we used *S. aureus* and anti-Tir antibodies to cluster HN-Tir-EspF_U_ fusions harboring various numbers of repeats in wild type and N-WASP-knockout FLCs. We then measured the fraction of cells that contained actin pedestals. Whereas in wild type FLCs, a single repeat resulted in pedestal formation levels of ∼45% (“R1”, Figure 8A, black bars [28]), this derivative generated no pedestals in N-WASP-knockout cells (Figure 8A, gray bars). (Note that the experiments with wild type and N-WASP-knockout cells were performed in parallel, but those using wild type cells were published previously [28] and are shown in Figure 8A for ease of comparison.) Clustering of Tir-EspF_U_ fusions harboring two to six repeats in N-WASP-knockout FLCs resulted in cellular pedestal formation efficiencies of approximately 50–55%, which is significantly less than the levels of 75–90% that were observed in wild type cells (Figure 8A; [28]).

To further investigate the relationship between number of repeat units and N-WASP-independent actin polymerization we sought to measure pedestal formation when EspF_U_ is present in the cytosol and Tir is independently translocated into the plasma membrane. Under these conditions, ∼90% of wild type cells expressing any GFP-EspF_U_ construct containing at least two repeats generated pedestals in response to infection with KC12 (Figure 8B; [28]). To similarly examine pedestal formation when EspF_U_ must act in concert with Tir in the absence of N-WASP, we infected GFP-EspF_U_-expressing N-WASP-knockout cells with KC12 (Figure S5). Only ∼50% of cells expressing the four- and six-repeat truncations generated pedestals, and just 15% of cells expressing the three-repeat derivative formed pedestals (Figure 8B). No pedestals were observed in cells expressing fewer than three repeats (Figure 8B). In addition, in cells expressing EspF_U_ derivatives harboring three or more repeats, pedestal formation was less efficient without N-WASP. Thus, N-WASP deficiency is associated with a more stringent requirement for multimeric EspF_U_ variants in order to trigger actin assembly, and even for multi-repeat EspF_U_ derivatives that do trigger assembly in N-WASP-knockout cells, the efficiency of pedestal formation was somewhat reduced.

### The WASP/N-WASP-binding region of EspF_U_ is required for N-WASP-independent actin pedestal formation

**Figure 9:**
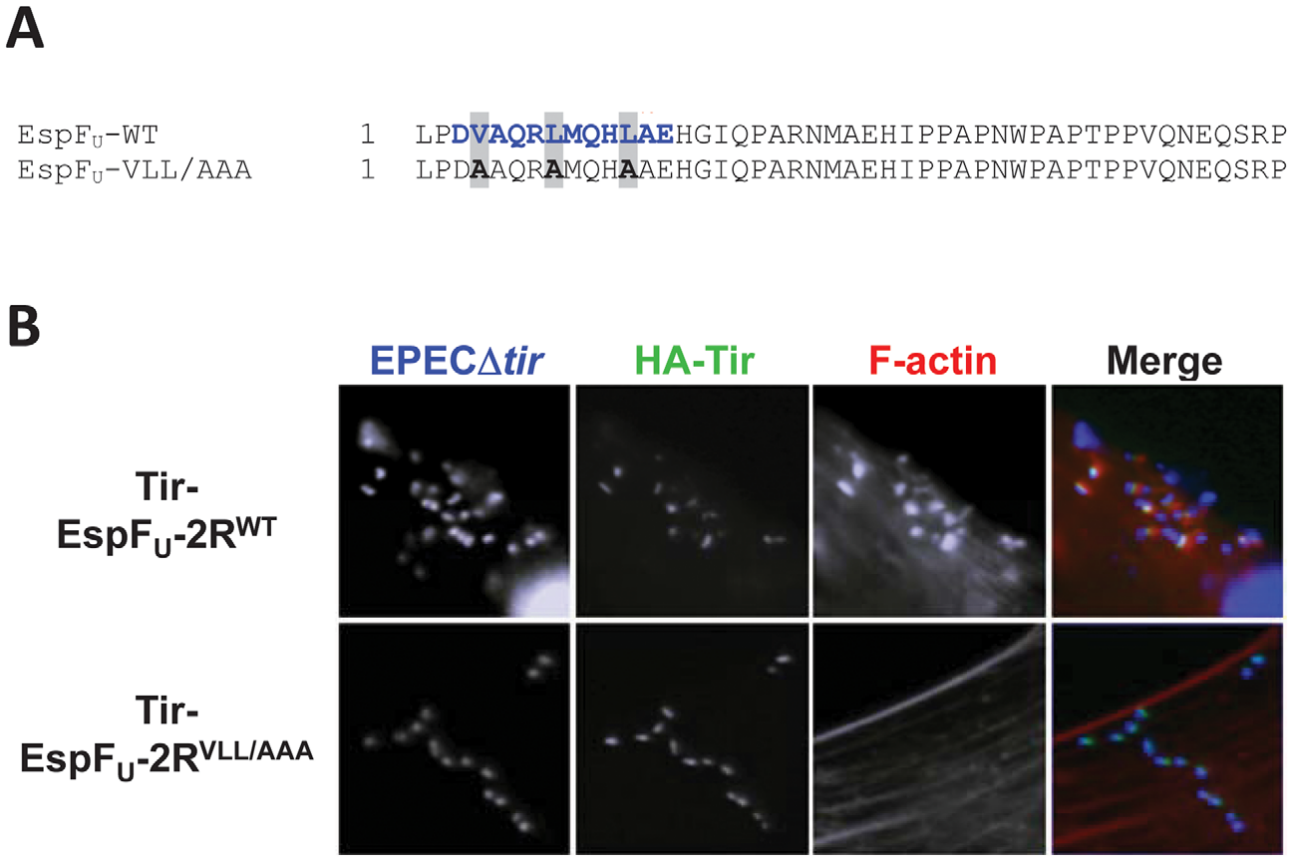
The WASP/N-WASP-binding region of EspF_U_ is required for N-WASP-independent actin pedestal formation. (**A**) Sequence alignment of an EspF_U_ repeat and the corresponding VLL/AAA mutant. The WASP/N-WASP-binding α-helix is colored in blue. (**B**) NW^−/−^ FLCs ectopically expressing HA-Tir-EspF_U_-2R^WT^ or HA-Tir-EspF_U_-2R^VLL/AAA^ fusions were challenged with an EPECΔ*tir* strain (which expresses intimin), and stained with DAPI (blue), anti-HA antibody (green) and Alexa568-phalloidin (red).

The EspF_U_ repeat contains an N-terminal region that, upon WASP binding, adopts an α-helical conformation that interacts with a hydrophobic groove in the GBD [26]. Thus, alanine substitution of three conserved hydrophobic residues in the EspF_U_ α-helix abolished N-WASP recruitment and actin assembly in mammalian cells [26]. To test whether this region of the EspF_U_ repeat plays an essential role in N-WASP-independent actin assembly, we constructed a Tir-EspF_U_ fusion comprising two EspF_U_ repeats that each harbored the V4A/L8A/L12A triple alanine substitution (referred to as VLL/AAA; Figure 9A). Tir-EspF_U_-2R^VLL/AAA^ and the corresponding wild type variant, Tir-EspF_U_-2R^WT^, were expressed in NW^−/−^ FLCs and clustered in the membrane using an EPEC strain that expresses intimin but not Tir or EspF_U_, and the cells stained with an anti-HA antibody to visualize the clustered fusion proteins and with phalloidin to stain F-actin (Figure 9B). Clustering of Tir-EspF_U_-2R^WT^ but not Tir-EspF_U_-2R^VLL/AAA^ induced robust pedestal formation under bound bacteria (Figure 9B, top row), indicating that the WASP/N-WASP-binding region of EspF_U_ is critical for N-WASP-independent actin pedestal formation.

## Discussion

N-WASP is required for actin pedestal formation by EHEC [12], and the observation that EspF_U_ directly binds and activates this nucleation-promoting factor provided an obvious explanation for this requirement. However, we now show that N-WASP is also important for an earlier step in actin pedestal formation, type III translocation of Tir and EspF_U_. We evaluated three properties of Tir that would reflect proper translocation into mammalian host cells. We assessed entry of Tir-Bla reporter proteins into the cytosol, quantified the ability of intimin-expressing bacteria to bind to primed host cells containing translocated Tir, and directly visualized the localization and clustering of Tir in the plasma membrane. These approaches each revealed that Tir translocation was diminished in N-WASP-knockout cells. The translocation defect was not restricted to Tir, because EHEC-mediated delivery of an EspF_U_-Bla fusion protein was also lower in N-WASP-knockout cells. Given that F-actin assembly promotes type III translocation of effectors by other pathogens [9], [10], it seems likely that the ability of N-WASP to promote actin assembly contributes to translocation by EHEC. Consistent with this possibility, translocation was significantly impaired by cytochalasin D or latrunculin A, which inhibit actin assembly, or by wiskostatin, an inhibitor of N-WASP [46].

These results raise the possibility that one of the functions of Tir- and EspF_U_-driven actin polymerization is to promote efficient translocation of one or more of the other 20–30 EHEC effectors. Interestingly, multiple pathogens encode type III secreted proteins that modify the actin cytoskeleton and have been shown to influence type III translocation. For example, the *Shigella* type III translocon protein IpaC stimulates Src recruitment and actin polymerization at sites of bacterial entry, and its inactivation diminishes type III translocation [10]. The *Yersinia* effectors YopE and YopT induce misregulation of Rho-family GTPases, inhibit signaling from these cytoskeletal regulators, and are postulated to temporally limit the phase of high efficiency type III translocation [9], [47], [48], [49], [50]. For EHEC, low levels of Tir translocation still occurred when actin polymerization was disrupted (Figure 1C), and multiple reports have demonstrated that EHEC mutants defective in pedestal formation are still capable of translocation [22], [39], [51], [52], indicating that actin assembly is not absolutely required for this process. This residual level of translocation may also explain the observation that for N-WASP^del/del^ cells, an independently derived N-WASP-deficient embryonic fibroblast cell line, Tir is translocated by an EHECΔe*spF_U_* mutant efficiently enough to recruit ectopically expressed GFP-EspF_U_ beneath sites of bacterial attachment [25].

Although it has now been shown that actin assembly promotes type III translocation by several pathogens, the specific function(s) of assembly is not clear. For EHEC, pedestal formation may increase the area of bacterium-host cell contact and/or the stability of bacterial binding, thereby enhancing effector translocation. Alternatively, type III translocation by several pathogens, including EPEC, is thought to occur at lipid microdomains [53], [54], [55], and it has been postulated that actin assembly may facilitate the recruitment of such domains to bound bacteria [9]. Interestingly, we found that although EPEC-mediated translocation of Tir into mammalian cells was somewhat delayed and diminished by N-WASP-deficiency, this defect was not large enough to have a discernable defect in intimin-mediated bacterial binding. The reasons for the lower N-WASP-dependence of translocation by EPEC are not known, but EPEC exhibits particularly robust type III secretion *in vitro* and generates pedestals more efficiently on cultured cells than does EHEC [35].

We utilized KC12/pEspF_U_, an EPEC strain engineered to express Tir_EHEC_ and EspF_U_, to more efficiently deliver EHEC Tir and EspF_U_ into N-WASP knock out cells. Surprisingly, these effectors were capable of generating actin pedestals with ultimately high efficiency: 95% of Tir foci beneath cell-bound KC12/pEspF_U_ were associated with pedestals in N-WASP-knockout cells after 5h infection. Thus, the defect in EHEC pedestal formation on N-WASP-knockout FLCs is apparently not due to an inability of Tir and EspF_U_ to stimulate actin polymerization once delivered to the mammalian cell.

The N-WASP-independent pathway of pedestal formation shares several parallels with pedestal formation in wild type cells. Tir and EspF_U_ are the only bacterial effectors required, since ectopic expression of the two proteins in N-WASP-knockout cells, followed by Tir clustering, was sufficient to induce localized actin assembly (data not shown). IRTKS, which has been shown to link EspF_U_ to Tir in N-WASP-proficient cells [24], [25], was recruited to sites of bacterial attachment on N-WASP-knockout cells. Moreover, the central role of the C-terminal cytoplasmic domain of Tir is to promote IRTKS-mediated recruitment of EspF_U_, because a Tir fusion protein in which this Tir domain is replaced by EspF_u_ was competent for triggering actin polymerization. Finally, the Arp2/3 complex, the actin nucleator that acts in conjunction with N-WASP, was recruited to sites of pedestal formation in N-WASP-knockout cells. Inactivation of Arp2/3 function blocked pedestal formation on both wild type and N-WASP-knockout cells, indicating that this nucleator is required for all pathways of pedestal formation.

EspF_U_ was unable to directly activate the Arp2/3 complex *in vitro*, suggesting that, in addition to recruiting and activating N-WASP, EspF_U_ also recruits and activates another regulator of actin assembly that directly or indirectly activates the Arp2/3 complex. Interestingly, a triple amino acid substitution in EspF_U_ that disrupts binding of EspF_U_ to WASP/N-WASP abolished pedestal formation in N-WASP-knockout cells, suggesting that the putative alternate regulator of actin assembly may recognize the same or an overlapping segment of EspF_U_. This finding is consistent with the hypothesis that one of the WASP-related nucleation promoting factors may participate in this pathway. However, GFP-tagged derivatives of WAVE2, WASH and WHAMM were not efficiently recruited to pedestals in N-WASP-knockout cells (Figure 7); although WAVE2 demonstrated a modest degree of colocalization with bacteria, pedestals were formed efficiently on N-WASP-deficient cells genetically depleted for WAVE2 (Figure S4). Interestingly, KC12/pEspF_U_ did not form pedestals on N-WASP^del/del^ cells, suggesting that this independently derived N-WASP-deficient cell line [13] may lack the putative alternate actin assembly factor (D.V., J.L., unpub. obs.). One can imagine several scenarios by which N-WASP^del/del^ cells might aid in the identification of the factor(s) responsible for driving actin polymerization in the absence of N-WASP, a finding that might provide new insights into the normal regulation of actin assembly in mammalian cells.

Notably, pedestal formation by this N-WASP-independent pathway occurred somewhat less efficiently than when both N-WASP and the putative factor are present, because clustering of Tir-EspF_U_ fusion protein generated pedestals 25–30% less efficiently on knockout cells than on wild type cells. In addition, pedestal formation on N-WASP-knockout cells showed a more stringent requirement for multiple EspF_U_ repeats. Whereas clustering of a single EspF_U_ repeat was sufficient to stimulate actin pedestals in the presence of N-WASP [26], [28], pedestal formation was only triggered upon clustering of two or more repeats in the absence of N-WASP. In addition, while two repeats are required to complement an *espF_U_*-deficient strain for pedestal formation on wild type FLCs [28], three repeats were required to detect pedestals in NW^−/−^ FLCs, and four or more repeats were required for maximal levels of complementation.

A correlation between the number of EspF_U_ repeats and stimulation of actin assembly, both *in vitro* and *in vivo*, has been observed previously [27], [28], [29], [39]. Assuming that at least three repeats are required for N-WASP-independent pedestal formation and that an N-WASP-independent pathway for actin assembly confers a selective advantage in nature, one would predict that the vast majority of EspF_U_ alleles found among *E. coli* isolates would carry at least three repeats. In fact, of 435 EPEC or EHEC strains in which *espF_U_* was detected by PCR, 433 (or >99.5%) of *espF_U_* alleles appeared, by length of the PCR product, to encode three or more proline-rich repeats [56]. Therefore, future characterizations of the N-WASP-independent mechanism of actin pedestal formation will enhance our understanding of the role of EspF_U_ in the survival, propagation, or pathogenesis of EHEC.

## Materials and Methods

### Bacteria, plasmids, and mammalian cell culture

All EHEC strains used in this study were derivatives of TUV93-0, a Shiga toxin-deficient version of the prototype 0157:H7 strain EDL933 [36]. The parental EPEC strain was the 0127:H6 prototype JPN15/pMAR7. EPEC KC12 [36], EPECΔ*tir* [36], EPECΔ*tir-eae* [36] and EHECΔ*eae* [57], EHECΔ*tir-eae* [57] were described previously. Non-pathogenic laboratory strains of *E.coli* harboring plasmids encoding EHEC or EPEC intimin (pInt) have also been described [33]. These strains were transformed with a separate plasmid for expression of GFP (a gift from A. Poteete). For beta-lactamase (Bla) translocation assays, plasmids pMB196 (pTir_EHEC_-Bla) and pMB200 (pEspF_U_-Bla) were constructed as follows: PCR products encoding Tir and EspF_U_ with their endogenous promoter were amplified with primers flanked with EcoRI and KpnI restriction sites, digested with the appropriate enzymes and cloned into the similarly digested plasmid pMM83 [58]. Plasmids used for expression of GFP-EspF_U_ and HA-Tir-EspF_U_ fusion proteins in mammalian cells were described previously [28]. For dominant negative transfection, the WCA domain of rat N-WASP was amplified by PCR and cloned into the KpnI-EcoRI sites of plasmid pKC425 [59]. All *E. coli* strains were grown in LB media at 37°C for routine passage. Before infection of mammalian cells, EHEC and EPEC were cultured in DMEM containing 100 mM HEPES pH 7.4, in 5% CO_2_ to enhance type III secretion. HeLa cells and FLCs were cultured in DMEM containing 10% FBS, 2mM glutamine and 50 µg/ml penicillin/streptomycin. Transfection of plasmid DNA was performed as previously described [52].

### RNA isolation and WASP RT-PCR

Total RNA from N-WASP-knockout cells was isolated using TRIzol reagent (Invitrogen). A first strand cDNA was synthesized using the M-MLV (Moloney Murine Leukemia Virus) Reverse Transcriptase (RT) (Invitrogen). Primers WASP_F (5′-GTGCAGGAGAAGATACAAAAAAGG-3′) and WASP_R (5′-GATCCCAGCCCACGTGGCTGACATG-3′) were used in a 40 cycle PCR reaction to detect WASP cDNA. cDNA from activated B cells, which express abundant WASP, was used as a positive control.

### Generation of N-WASP knockout/WAVE2 knockdown cells

Murine WAVE2 sequence (5′-GAGAAAGCATAGGAAAGAA-3′) was cloned into the Hpa1 and Xho1 sites of plasmid Lentilox 3.7 (pLL 3.7) to generate WAVE2 RNAi stem loops. The virus was packaged into 293T cells using a four-plasmid system and was collected 48 hours after transfection. To knockdown WAVE2, N-WASP-deficient cells were transformed with the lentivirus containing the WAVE2 RNAi stem loop. Knockdown efficiency was evaluated by western blot of the transformed N-WASP KO cells using anti-WAVE2 antibody (Santa Cruz Biotechnology). N-WASP-deficient cells transformed with the empty lentiviral vector were used as control.

### EHEC and EPEC Infections

Infections of HeLa cells and FLCs with EHEC and EPEC strains were performed as described in earlier work [36]. To evaluate intimin-mediated bacterial attachment in priming-and-challenge assays, FLCs were infected (“primed”) for 3h with EPECΔ*eae*, or EPECΔ*tir*-*eae* mutant harboring plasmids encoding HA-Tir_EPEC_ or HA-Tir_EHEC_, or for 5h with EHECΔ*eae*, or EHECΔ*tir*-*eae* mutant harboring plasmids encoding HA-Tir_EPEC_ or HA-Tir_EHEC_. These strains translocate Tir but do not form pedestals, and were removed from the cell monolayers after gentamicin treatment and washing. The primed cells were then infected (“challenged”) for 1h with non-pathogenic laboratory strains of *E.coli* harboring plasmids encoding either EHEC or EPEC intimin (pInt) and a plasmid that expresses GFP. A bacterial binding index, defined as the percentage of cells with at least five bound GFP-expressing bacteria, was determined microscopically (Figure 2).

### Bla fusion translocation assays

To determine the translocation index of Bla fusions into NW^+/+^ or NW^−/−^ FLCs, cells were infected for 6 hours with EPEC or EHEC strains harboring Tir-Bla or EspF_U_-Bla fusions. Infected monolayers were washed with PBS and incubated for 1–2 hours at room temperature after addition of CCF2-AM (Invitrogen) supplemented medium. CCF2-AM treated cells were fixed and analyzed microscopically using a 20× objective. The percentage of blue cells, reflecting effector translocation, was estimated for 10–20 fields per experiment. For studies involving chemical inhibitors of actin assembly, DMSO, wiskostation or cytochalasin D (Sigma) was added 1 hour before infection. To determine the effect of wiskostatin on effector translocation, HeLa cells were infected with EHEC/pEspF_U_-Bla at a density of 2×10^7^ bacteria/well in DMEM containing either DMSO or 6 µM wiskostatin. Plates were spun at 200 RCF for 5 minutes and then incubated at 37°C in 5% CO_2_ for 90 minutes. Cells were washed twice with PBS, overlaid with 100 µl of CCF2/AM loading solution in PBS, and then incubated for two hours at room temperature. Plates were transferred to a Synergy 2 microplate reader (BioTek) and excited at 400 nm (10-nm band-pass) and the emission signal was read at 460 nm (40-nm band-pass) and 528 nm (20-nm band-pass). After subtracting out background, the 460/528 nm ratio was calculated to determine the level of effector translocation. Upon treatment with cytochalasin D, HeLa cells were infected with EHEC/pHA-Tir and samples processed for detection of Tir foci and F-actin pedestals by immunofluorescence microscopy, as described below.

### Immunofluorescence microscopy

Infected cells were fixed in 2.5% paraformaldehyde for 35 minutes and permeabilized with 0.1% Triton-X-100 in PBS as described previously [36]. Bacteria were visualized using DAPI (1 µg/ml; Sigma), and F-actin was detected using 4 U/ml Alexa568-phalloidin (Invitrogen). To visualize HA-Tir derivatives, EspF_U_-myc, IRTKS, or IRSp53, cells were treated with mouse anti-HA tag mAb HA.11 (1∶500; Covance), mouse anti-myc 9E10 mAb (1∶250; Santa Cruz Biotechnology), or mouse anti-IRTKS mAb (1∶100; Novus Biologicals) prior to treatment with Alexa488-conjugated goat anti-mouse antibody (1∶150; Invitrogen). To visualize the Arp2/3 complex, cells were treated with rabbit anti-Arp3 antibodies (1∶150; gift from R. Isberg, Tufts University) prior to treatment with Alexa488-conjugated goat anti-rabbit antibody (1∶150; Invitrogen). To determine the pedestal formation efficiency of EPEC variants expressing HA-tagged Tir (Figure 3D), the percentage of sites of translocated Tir (HA-Tir foci) that were associated with intense F-actin staining in FLCs were counted. To determine the pedestal efficiency in mammalian cells expressing HA-Tir-EspF_U_ fusions or GFP-EspF_U_ derivatives, which were identified by anti-HA or GFP fluorescence, the percentage of cells harboring at least 5 adherent *S. aureus* particles (Figure 8A) or KC12 bacteria (Figure 8B) that were associated with actin pedestals was quantified. At least 50 cells were examined per sample. Cells expressing extremely high fluorescence levels of EspF_U_ were refractory to pedestal formation and were not included in these analyses.

### Pyrene-actin assembly assays

In vitro actin polymerization assays were performed using 500 nM EspF_U_ derivative, 2.0 µM actin (7% pyrene-labeled) and 20 nM recombinant Arp2/3 complex, in the presence of 20 nM N-WASP/WIP complex or not, and polymerization was measured as described previously [28].

### Preparation of cell lysates and immunoblotting

Cells were collected in PBS plus 2mM EDTA, washed with PBS, and lysed in lysis buffer [50mMTris-HCl, pH 8.0, 150mM NaCl, 1% Triton X-100, 1mM Na_3_VO_4_, 1mM PMSF, and 10µg/mL each of aprotinin, leupeptin, and pepstatin (Sigma)] before mixing with sample buffer. Samples were boiled for 10 min, separated by 10% SDS/PAGE, and transferred to PVDF membranes. Membranes were blocked in PBS containing 5% milk before treatment with anti N-WASP, anti-WASP (Santa Cruz Biotechnology), anti-Nck1 (Upstate), anti-Arp3, or anti-tubulin DM1A (Thermo Scientific) antibodies. Following washes, membranes were treated with secondary antibodies and developed [36].

## Supporting Information

Figure S1 Translocation of Tir_EHEC_ and EspF_U_ by EHEC is impaired in the absence of N-WASP-mediated actin polymerization. **(A)** Tir_EHEC_ fused to TEM-1 β-lactamase is able to complement an EHEC *tir* mutant for pedestal formation. HeLa cells were infected with EHECΔ*tir*(pTir-Bla) and monoloyers were examined after staining with DAPI to detect bacteria (blue) and Alexa568-phalloidin to visualize F-actin (red). **(B)** Translocation of Tir-Bla and EspF_U_-Bla fusions into fibroblast-like cells. NW^+/+^ and NW^−/−^ fibroblast-like cells were infected for 3 hours with wild type EHEC harboring plasmids encoding Tir-Bla or EspF_U_-Bla fusions. Translocation of the fusion proteins into host cells was measured by detecting cleavage of the β-lactamase FRET reporter CCF2-AM, which results in a change in fluorescent emission of cells from green (absence of detectable Tir-Bla) to blue (presence of Tir-Bla). **(C)** The N-WASP inhibitor wiskostatin impairs translocation of an EspF_U_-Bla fusion into HeLa cells. HeLa cells were treated with DMSO or wiskostatin (6 µM) and infected with EHEC/pEspF_U_-Bla for 3.5 hours. Translocation of the fusion protein into host cells was measured by detecting cleavage of the β-lactamase FRET reporter CCF2-AM. The level of effector translocation was expressed as the ration of OD_460nm_/OD_528nm_.
(1.13 MB TIF)

Figure S2 Translocation of a Tir-Bla or EspF_U_-Bla fusion by EPEC KC12 in NW^+/+^ and NW^−/−^ cells. Monolayers were infected for 3 hours with KC12 expressing the Tir_EHEC_-Bla or EspF_U_-Bla fusion, incubated with CCF2-AM, and fixed. Translocation was measured by detecting cleavage of CCF2-AM, which results in a change in fluorescent emission of cells from green (absence of detectable effector-Bla) to blue (presence of effector-Bla) [31]. The percentage of blue cells was scored visually by fluorescent microscopy to determine the translocation index. Shown is the mean ± SD of three experiments.
(0.29 MB TIF)

Figure S3 The Arp2/3 complex is critical for EHEC-induced actin pedestal formation on HeLa cells. The Arp2/3 complex subunits Arp3+ARPC4 were knocked down in HeLa cells, as described previously [60]. Cells were transfected with control siRNA, GADPH siRNA or Arp3+ARPC4 siRNAs and infected with EHEC, fixed, and stained with DAPI to detect bacteria and Alexa568-phalloidin to detect F-actin. The percentage of cells with actin pedestals was determined visually by fluorescence microscopy. Shown is the mean ± SD of three experiments.
(1.04 MB TIF)

Figure S4 EspF_U_ does not directly activate the Arp2/3 complex *in vitro*. Polymerization of pyrene-labeled actin (2 µM; 7% pyrene-labeled) was measured over time in the presence of Arp2/3 complex (20 nM) and purified recombinant EspF_U_ derivatives (500 nM). The control reaction was performed in the presence of 20 nM N-WASP/WIP complex. F-actin fluorescence was expressed in arbitrary units (AU).
(0.32 MB TIF)

Figure S5 EPEC KC12 triggers pedestal formation in N-WASP knockout cells ectopically expressing the C-terminal region of EspF_U_. NW^−/−^ fibroblast-like cells ectopically expressing GFP or GFP fused to the C-terminal repeats of EspF_U_ (GFP-EspF_U_-[R1-6]) were infected with EPEC KC12, an EPEC-derived strain that expresses EHEC Tir from the chromosome [36]. Monolayers were fixed and examined after staining with DAPI to detect bacteria (blue), anti-myc antibody to detect GFP-myc fusions (green) and Alexa568-phalloidin to visualize F-actin(red). EspF_U_ is efficiently recruited to sites of KC12 attachment and cooperate with Tir_EHEC_ to induce N-WASP-independent actin polymerization.
(0.39 MB TIF)
