# Supplementary figures and images for: Enterohemorrhagic E. coli Requires N-WASP for Efficient Type III Translocation but Not for EspFU-Mediated Actin Pedestal Formation
Source: PLoS Pathog. 2010 Aug 19;6(8):e1001056. doi: 10.1371/journal.ppat.1001056 (PMC2924363; doi:10.1371/journal.ppat.1001056)

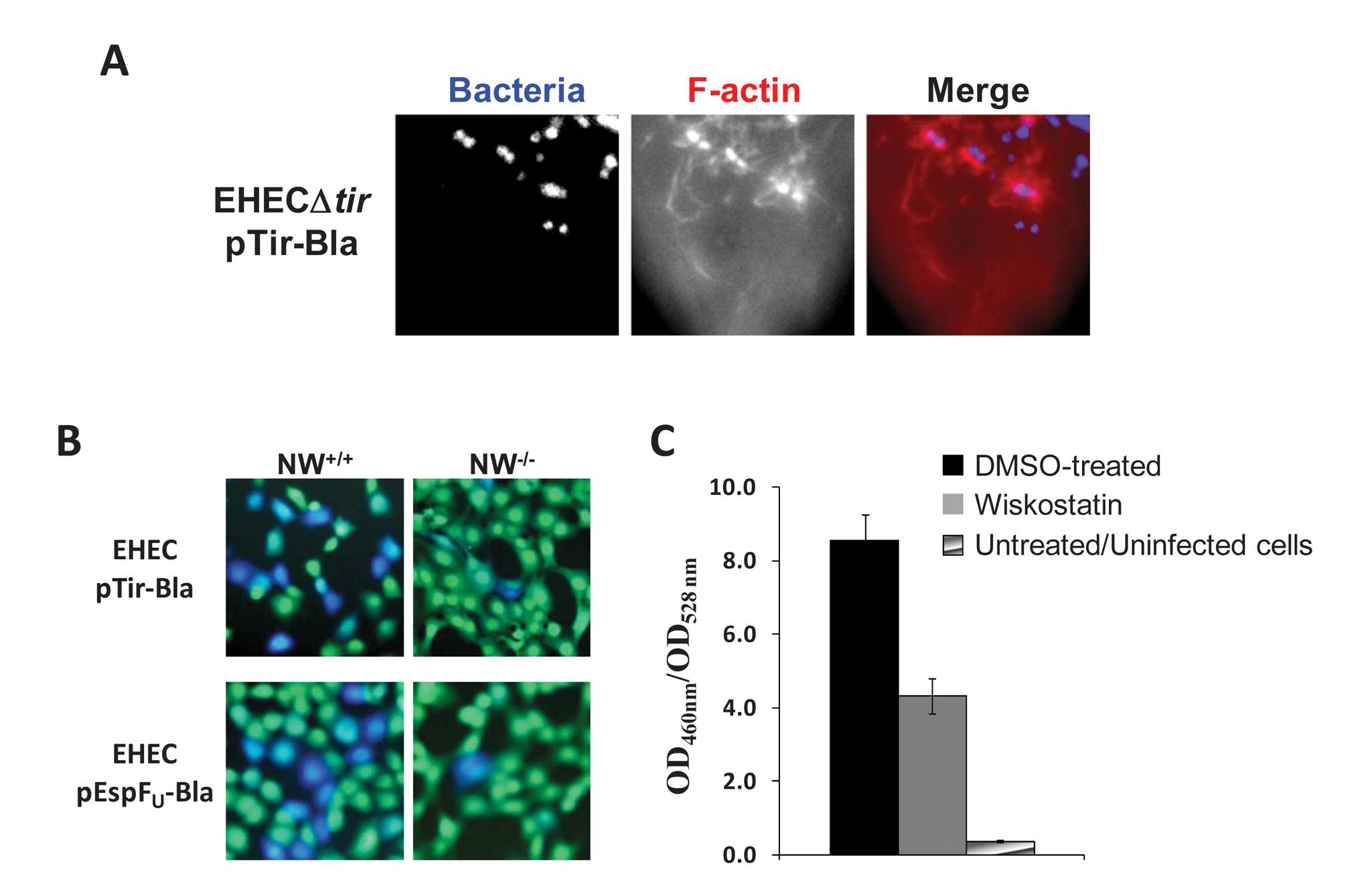

Supplement: Figure S1 — Translocation of TirEHEC and EspFU by EHEC is impaired in the absence of N-WASP-mediated actin polymerization. (A) TirEHEC fused to TEM-1 β-lactamase is able to complement an EHEC tir mutant for pedestal formation. HeLa cells were infected with EHECΔtir(pTir-Bla) and monoloyers were examined after staining with DAPI to detect bacteria (blue) and Alexa568-phalloidin to visualize F-actin (red). (B) Translocation of Tir-Bla and EspFU-Bla fusions into fibroblast-like cells. NW+/+ and NW−/− fibroblast-like cells were infected for 3 hours with wild type EHEC harboring plasmids encoding Tir-Bla or EspFU-Bla fusions. Translocation of the fusion proteins into host cells was measured by detecting cleavage of the β-lactamase FRET reporter CCF2-AM, which results in a change in fluorescent emission of cells from green (absence of detectable Tir-Bla) to blue (presence of Tir-Bla). (C) The N-WASP inhibitor wiskostatin impairs translocation of an EspFU-Bla fusion into HeLa cells. HeLa cells were treated with DMSO or wiskostatin (6 µM) and infected with EHEC/pEspFU-Bla for 3.5 hours. Translocation of the fusion protein into host cells was measured by detecting cleavage of the β-lactamase FRET reporter CCF2-AM. The level of effector translocation was expressed as the ration of OD460nm/OD528nm. (1.13 MB TIF) [file ppat.1001056.s001.tif]

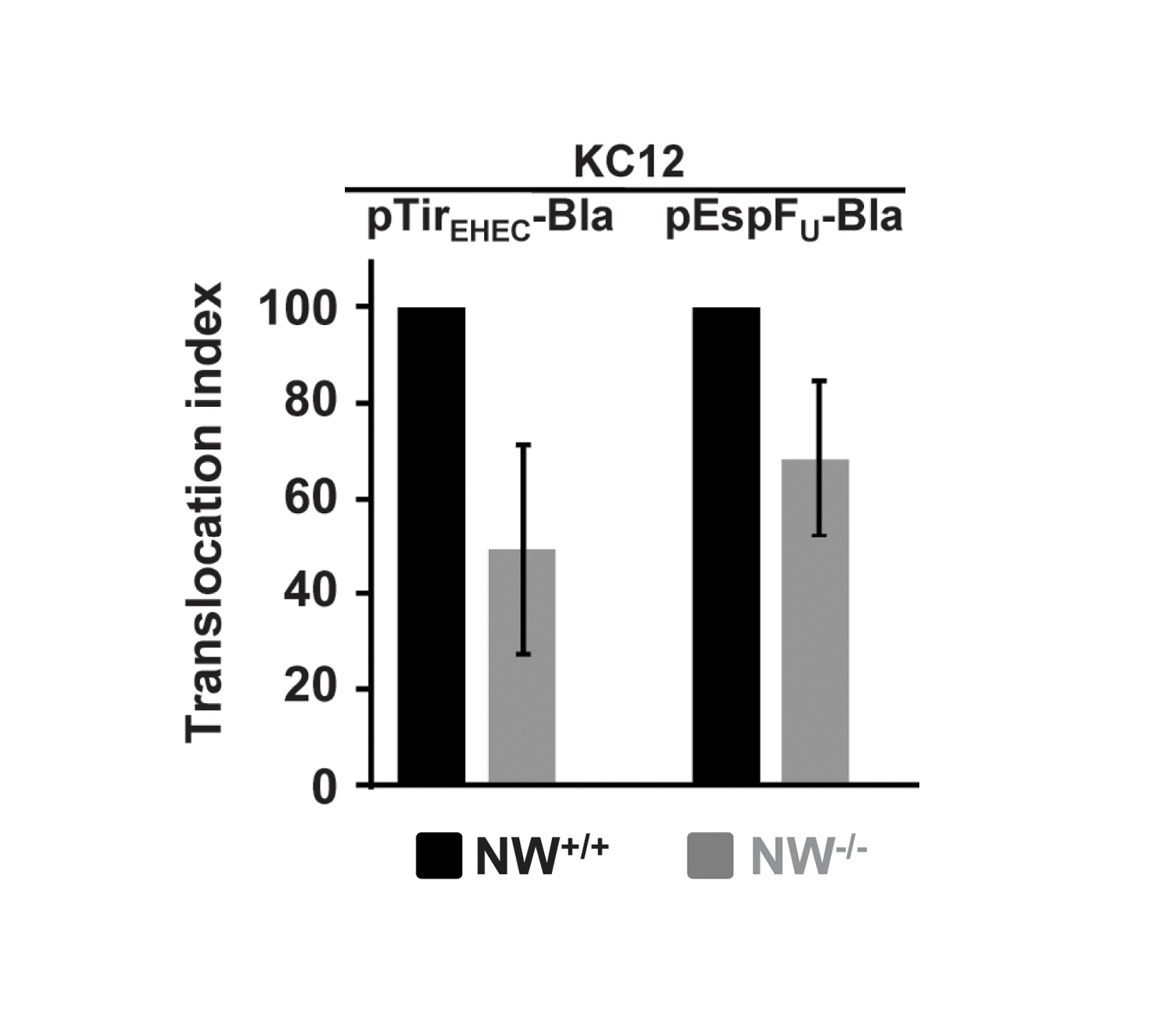

Supplement: Figure S2 — Translocation of a Tir-Bla or EspFU-Bla fusion by EPEC KC12 in NW+/+ and NW−/− cells. Monolayers were infected for 3 hours with KC12 expressing the TirEHEC-Bla or EspFU-Bla fusion, incubated with CCF2-AM, and fixed. Translocation was measured by detecting cleavage of CCF2-AM, which results in a change in fluorescent emission of cells from green (absence of detectable effector-Bla) to blue (presence of effector-Bla) [31]. The percentage of blue cells was scored visually by fluorescent microscopy to determine the translocation index. Shown is the mean ± SD of three experiments. (0.29 MB TIF) [file ppat.1001056.s002.tif]

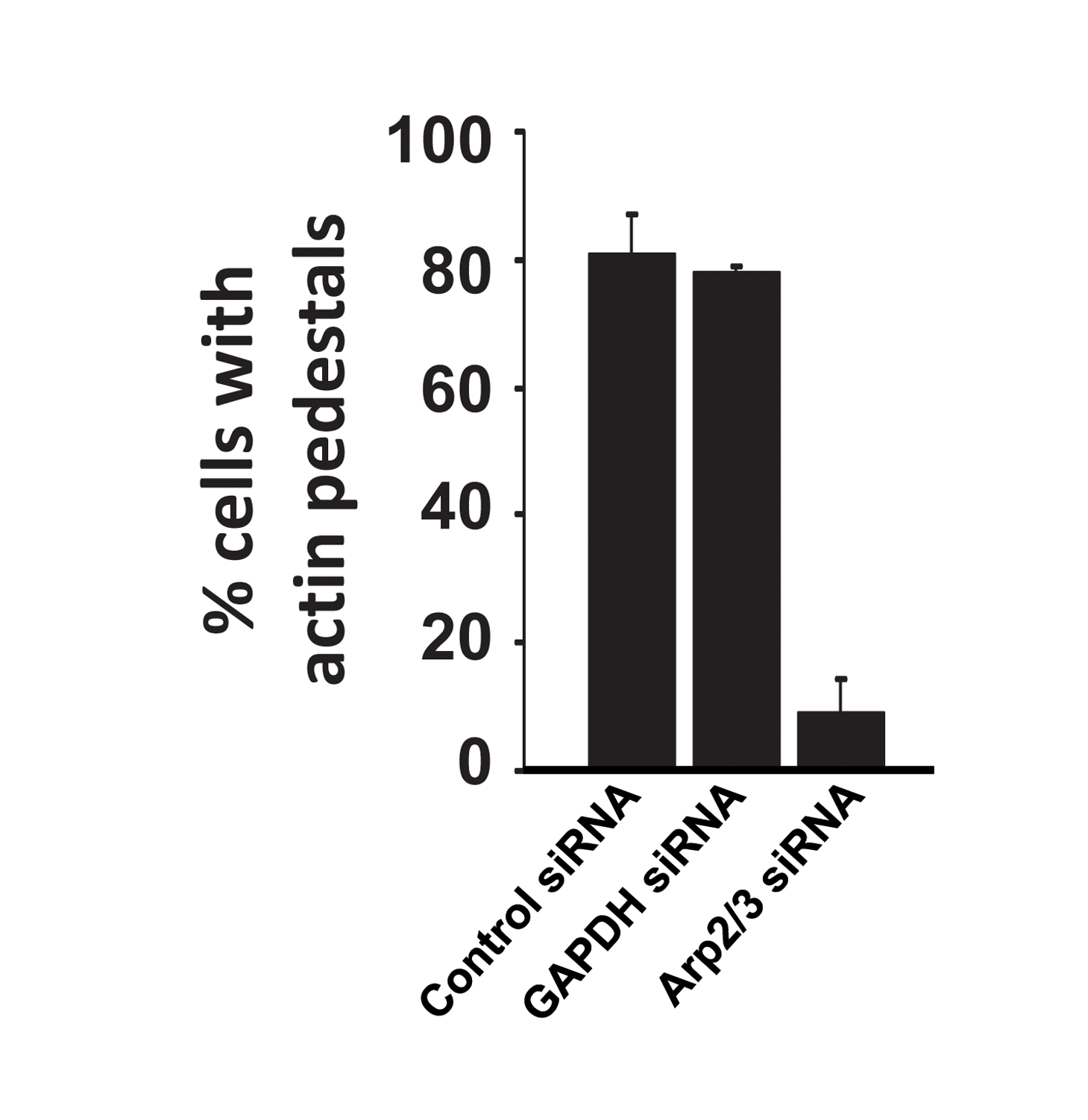

Supplement: Figure S3 — The Arp2/3 complex is critical for EHEC-induced actin pedestal formation on HeLa cells. The Arp2/3 complex subunits Arp3+ARPC4 were knocked down in HeLa cells, as described previously [60]. Cells were transfected with control siRNA, GADPH siRNA or Arp3+ARPC4 siRNAs and infected with EHEC, fixed, and stained with DAPI to detect bacteria and Alexa568-phalloidin to detect F-actin. The percentage of cells with actin pedestals was determined visually by fluorescence microscopy. Shown is the mean ± SD of three experiments. (1.04 MB TIF) [file ppat.1001056.s003.tif]

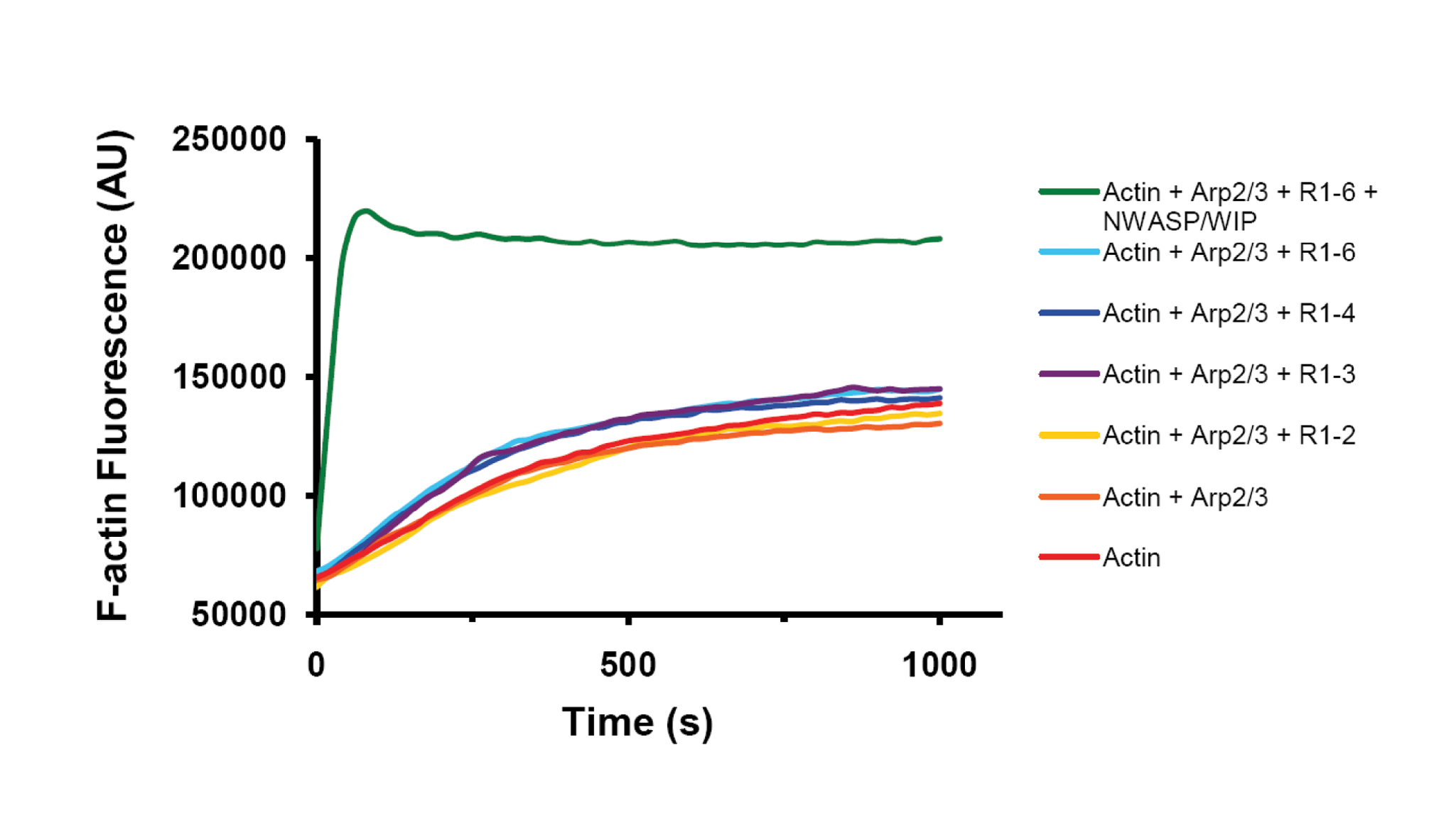

Supplement: Figure S4 — EspFU does not directly activate the Arp2/3 complex in vitro. Polymerization of pyrene-labeled actin (2 µM; 7% pyrene-labeled) was measured over time in the presence of Arp2/3 complex (20 nM) and purified recombinant EspFU derivatives (500 nM). The control reaction was performed in the presence of 20 nM N-WASP/WIP complex. F-actin fluorescence was expressed in arbitrary units (AU). (0.32 MB TIF) [file ppat.1001056.s004.tif]

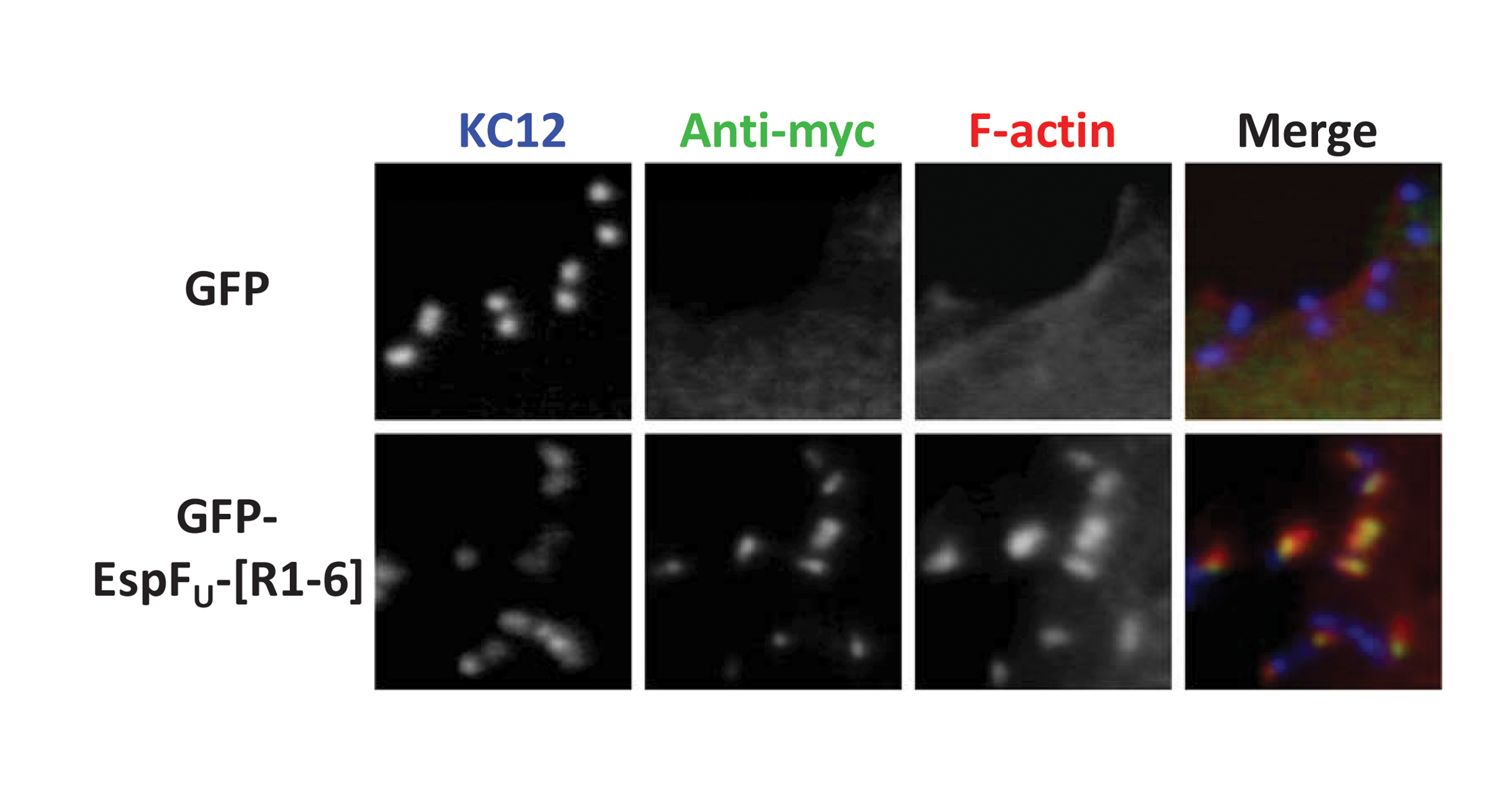

Supplement: Figure S5 — EPEC KC12 triggers pedestal formation in N-WASP knockout cells ectopically expressing the C-terminal region of EspFU. NW−/− fibroblast-like cells ectopically expressing GFP or GFP fused to the C-terminal repeats of EspFU (GFP-EspFU-[R1-6]) were infected with EPEC KC12, an EPEC-derived strain that expresses EHEC Tir from the chromosome [36]. Monolayers were fixed and examined after staining with DAPI to detect bacteria (blue), anti-myc antibody to detect GFP-myc fusions (green) and Alexa568-phalloidin to visualize F-actin(red). EspFU is efficiently recruited to sites of KC12 attachment and cooperate with TirEHEC to induce N-WASP-independent actin polymerization. (0.39 MB TIF) [file ppat.1001056.s005.tif]
